# Quantifying mass transport limitations in a microfluidic CO_2_ electrolyzer with a gas diffusion cathode

**DOI:** 10.1038/s42004-024-01122-5

**Published:** 2024-03-05

**Authors:** Venu Gopal Agarwal, Sophia Haussener

**Affiliations:** Laboratory of Renewable Energy Science and Engineering, EPFL, Station 9, Lausanne, 1015 Switzerland

**Keywords:** Energy modelling, Renewable energy, Electrocatalysis

## Abstract

A gas diffusion electrode (GDE) based CO_2_ electrolyzer shows enhanced CO_2_ transport to the catalyst surface, significantly increasing current density compared to traditional planar immersed electrodes. A two-dimensional model for the cathode side of a microfluidic CO_2_ to CO electrolysis device with a GDE is developed. The model, validated against experimental data, examines key operational parameters and electrode materials. It predicts an initial rise in CO partial current density (PCD), peaking at 75 mA cm^−2^ at −1.3 V vs RHE for a fully flooded catalyst layer, then declining due to continuous decrease in CO_2_ availability near the catalyst surface. Factors like electrolyte flow rate and CO_2_ gas mass flow rate influence PCD, with a trade-off between high CO PCD and CO_2_ conversion efficiency observed with increased CO_2_ gas flow. We observe that a significant portion of the catalyst layer remains underutilized, and suggest improvements like varying electrode porosity and anisotropic layers to enhance mass transport and CO PCD. This research offers insights into optimizing CO_2_ electrolysis device performance.

## Abstract (web summary)

CO_2_ electrolyzers with gas diffusion electrodes take advantage of improved mass transport of gaseous CO_2_ to the catalyst surface to afford increased current densities, but the complex and coupled multi-phase processes occurring inside the electrolysis device are not fully understood. Here, the authors use a two-dimensional volume-averaged model of the cathode side of a microfluidic CO_2_ to CO electrolysis device with a gas diffusion electrode and find that under high cathodic potential, the catalyst layer is prone to forming H_2_ and CO bubbles, mirroring observed experimental electrode instability.

## Introduction

Electrochemical CO_2_ reduction (CO_2_ER) using renewable electricity is a promising approach to replace fossil fuels and petrochemical-based platform chemicals with renewable alternatives^1–3^. CO_2_ electrolyzers usually employ either planar full metal electrodes in liquid electrolyte^4,5^ or porous gas diffusion electrodes (GDEs) in two-phase (liquid-gaseous) conditions^6,7^. Planar electrode-based CO_2_ electrolyzer utilizes dissolved CO_2_ in the aqueous electrolyte as a reactant. Studies involving such reactor designs provide fundamental knowledge in terms of kinetics of CO_2_ electroreduction of a particular catalyst i.e., allow for catalyst development and screening^8^. However, such an architecture is infeasible for industrial applications^9^ due to low CO_2_ reduction current density limited by poor mass transport of CO_2_ to the cathode owing to its low solubility and diffusivity in the electrolyte^10^. GDEs based flow cells partially overcome the limitations of planar flow cells by delivering CO_2_ closely to the cathode catalyst site directly in the gas phase through a porous gas diffusion layer, thereby enabling fast CO_2_ mass transfer^11^. As a result, a sufficiently higher concentration of CO_2_ is maintained close to the catalyst surface (compared to planar electrodes), achieving high rates of CO_2_ conversion, a requirement for commercial applications^12,13^.

The performance of a CO_2_ electrolyzer is acutely sensitive to the chemical environment near the electrocatalyst^14^ which is dictated by the coupled transport of gases, liquids, and ions along with the kinetics of various (electro)chemical reactions and the transport of electrons in the porous solid conductor, all taking place simultaneously in the GDE. To be able to control the local microenvironment and improve the performance of a CO_2_ electrolyzer, it is imperative to understand these concurrently occurring multi-physical processes inside the porous electrodes. Complex coupling between these processes along with their multi-scale nature makes it challenging to probe them experimentally and quantify their rates. Physics-based continuum modeling, on the other hand, is well suited to rationalize the observed behavior of a CO_2_ electrolyzer as a function of design, material, and operating parameters by resolving the local chemical environment. Such physical models, when compared with experimental studies, can provide fundamental physical insight that can accelerate the design of an efficient and industrially relevant CO_2_ electrolyzer^11^.

Continuum models at the macro-scale treat the porous electrode as a homogeneous, volume-averaged continuum to describe the transport and reaction processes that occur across the entire domain of the porous electrode^11^. Several modeling studies^15–19^ have helped to gain understanding of the influence of operating parameters (such as applied cathode potential and CO_2_ gas-flow rates) and GDE design parameters (such as cathode catalyst layer loading, CL and GDL porosities and conductivities) on CO_2_ reduction catalytic activity and selectivity. Existing GDE models either consider only a through-plane dimension (1D model)^15–17^ or through and in-plane directions (2D model)^18,19^. 1D models are the simplest models and have been shown to capture the potential and species concentration gradients across the catalyst and the transport layers relatively well. One of the most comprehensive 1D GDE model for CO_2_ electroreduction to CO has been developed by Weng et al.^15^. Their model demonstrated and explained the enhanced mass-transfer of reactant CO_2_ through the pores of the GDE which resulted in two orders of magnitude higher CO_2_ reduction current density compared to a planar cathode.

Although these 1D models provide important insights into the effect of process parameters, design, and material properties on the performance of the GDE, they cannot resolve the in-plane CO_2_ concentration gradient that exists in the gas flow channel. Consequently, in-plane spatial activity and selectivity gradients of electrochemical CO_2_ reduction in the GDE is also not captured. Only recently, few 2D GDE models were developed for CO_2_ electroreduction to CO on a silver surface^18,19^. The models simulated the in-plane flow of CO_2_ in the gas flow channel and highlighted a trade-off between high and low CO_2_ flow rates: high flow rates enabled higher CO_2_ reduction rates but lower single-pass CO_2_ conversion efficiency and vice-versa. These results corroborate the importance of incorporating the in-plane direction for developing more refined and sophisticated GDE models for CO_2_ electroreduction. However, previously developed 2D GDE models are restricted by assumptions limiting their applicability to a narrow range of operating conditions. In particular, these models assume the water-dissociation reaction to be at equilibrium (neglecting water-dissociation kinetics) and a constant value of Henry’s constant (neglecting Sechenov effect). In addition, the models do not account for the contribution of electro-migration to the flux of electrolytic species in the CL and electrolyte channel. These assumptions no longer remain valid at high operating current densities and can therefore introduce inaccuracies in the predicted electrode performance preventing experimental validation. Recently, Blake et al.^20^ addressed these mentioned model assumptions and developed a more comprehensive 2D GDE model and analyzed the inhomogeneities in the catalyst channel limiting the up-scaling of the CO_2_ electrolyzer. However, anisotropy and heterogeneity in the system still is not accurately considered, CL transport properties are based on simple analytical expressions that do not represent the realistic characteristics^21^, and the effect of product evolution (H_2_ and CO_2_) is neglected.

We develop a comprehensive 2D volume-average model of a GDE integrated with electrolyte and CO_2_ flow channel to predict the influence of several key operating parameters (applied cathode potential, electrolyte and CO_2_ gas flow rate) and electrode (CL) material properties on the system’s performance. The catalyst layer is made of silver nanoparticles which selectively reduce CO_2_ to CO. The mass transport limitations for such an electrolyzer design are identified and appropriate guidelines are suggested to overcome them.

## Results and discussion

We analyze the effect of various operating conditions (applied cathode potential, electrolyte flow rate, and CO_2_ gaseous flow rate) and electrode (CL) properties (electrode material, porosity, anisotropy in diffusivity correction) on the macroscopic performance parameters of the cathode GDE (CO PCD, CO faradaic efficiency (FE), CO_2_ conversion and consumption efficiencies). The predicted performance parameters are analyzed in terms of the locally, 2D-resolved concentration distributions of the different species. While macroscopic performance parameters can relatively easily be measured, there is little experimental data available on locally resolved characteristics, pointing to the value of our modeling study.

### Effect of operating conditions

#### Effect of applied cathode potential

**Fig. 1:**
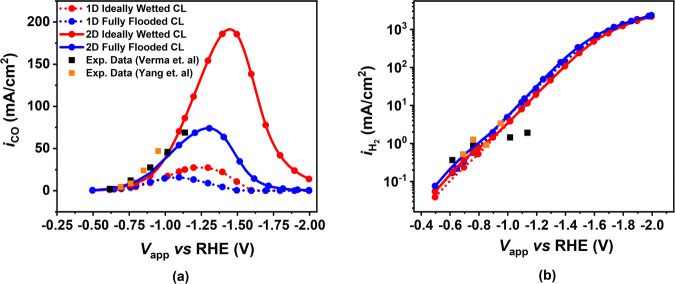
Effect of applied cathode potential on CO and H_2_ partial current density (PCD). **a** CO and **b** H_2_ PCD as a function of applied cathode potential for 1D and 2D modeling cases, for both ideally wetted (red) and fully flooded models (blue) of the Catalyst Layer (CL), and all compared with experimental observations from Verma et al. (black squares)^13^ and Yang et al. (orange squares)^22^. The dotted and solid lines are for 1D and 2D case, respectively. The model parameters correspond to those mentioned in Supplementary Tables 1 to 5 in the SI.

**Fig. 2:**
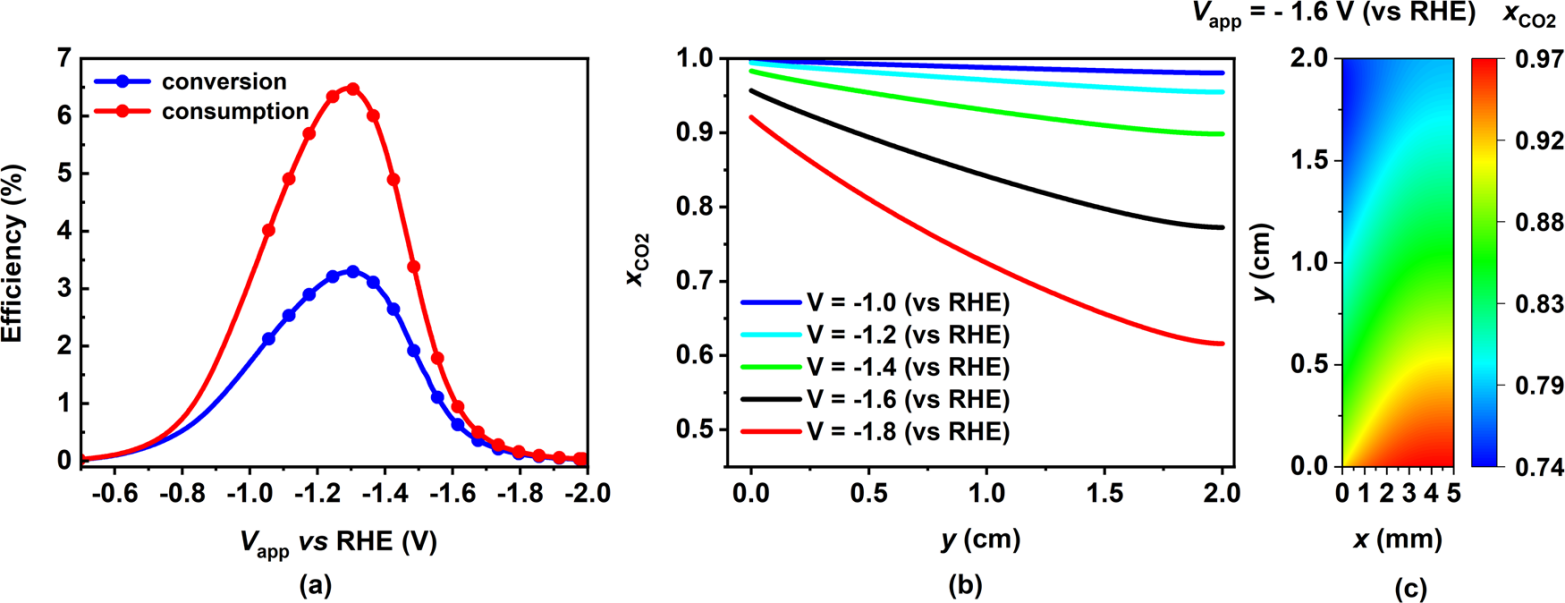
Effect of applied cathode potential on CO_2_ conversion and consumption efficiency along with CO_2_ mole fraction in the gas channel (GC). **a** Variation of CO_2_ conversion (blue) and consumption efficiencies (red) as a function of applied cathode potential. **b** Variation of averaged CO_2_ mole fraction along the length of the electrode in the GC for five different applied cathode potentials between −1.0 V to −1.8 V vs RHE (blue to red curves). The mole fractions are averaged along the thickness of the GC. **c** Contour of CO_2_ mole fraction in the GC for *V*_app_ = −1.6 V. The model parameters correspond to the base case (F.F CL) mentioned in Supplementary Tables 1 to 5 in the SI.

Figure 1 displays the variation of CO and H_2_ PCDs with applied cathode potential obtained for two different CL wetting scenarios (ideally wetted - I.W - and fully flooded - F.F) and two different model dimensionalities (1D and 2D). The predictions are compared with experimental measurements from Verma et al.^13^ and Yang et al.^22^. The 1D GDE model is found to underpredict the CO PCD which can be attributed to its inability to capture the heterogeneity in the gaseous mixture composition entering the GDL and its use of an analytical expression to estimate the flux of the electrolyte species entering the CL^15^. The 2D GDE model shows a better agreement (*R*^2^ value of 85% and 93.8%, respectively, for I.W and F.F case) with experimental observations compared to the 1D cases (*R*^2^ value −11% and −50%, respectively, for I.W and F.F case). This demonstrates the importance of incorporating a lateral flow direction and the induced variation in concentrations along the channels for a more realistic representation of the physical phenomena occurring inside a CO_2_ electrolysis device with GDE. The results predict that in the 1D and 2D scenarios, the ideally wetted CL exhibits higher CO PCD across all applied potentials when compared to fully flooded CL configurations. This observation is attributed to a more efficient transport of the CO_2_ reactant through the ideally wetted CL. In this configuration, CO_2_ can readily diffuse through the CL in its gaseous phase, which allows for faster transport and a higher concentration within the CL. In the fully flooded scenario, CO_2_ initially undergoes a phase transfer at the interface between the GDL and the CL. Subsequently, it must diffuse through the CL in the aqueous phase, where it has a lower concentration and diffusivity. This hindered transport of CO_2_ as a reactant results in reduced accessibility of the active catalyst sites within the CL, consequently leading to a lower CO PCD. Our 2D GDE model still incorporates few simplistic assumptions. For instance, the kinetics of COER on silver nano-particles were assumed to be the same as those on metallic silver foil used in planar electrode experiments^15^. The fully flooded model is utilized as the baseline case for all the subsequent analyses showcased in Fig. 2 through Fig. 10. Unless explicitly mentioned, all model parameters and operating conditions correspond to the values outlined in Supplementary Tables 2 to 5 within the SI.

Both 1D and 2D GDE models exhibit a common characteristic of CO PCD evolution with increased applied potential. Initially, the growth is exponential due to a kinetically controlled regime, but it slows down due to mass transport limitations and eventually reaches a peak current density. Beyond this peak, the CO PCD decreases. The mass transport limitation is caused by a continuous decrease in the CO_2_ concentration in the CL with increased applied potential. The consumption of aqueous CO_2_ from the electrolyte is higher than its replenishment from the gaseous phase via the GC. CO_2_ gets consumed through electrochemical and homogeneous reactions (Eq. (16) and Eq. (24)). Further, the rate of CO_2_ phase transfer reaction decreases with increasing ionic strength of the electrolyte (Sechenov effect), causing an additional bottleneck in the replenishment of aqueous CO_2_ from the CO_2_ supplied in the gaseous phase. When CO PCD reaches its peak, the CO_2_ concentration has decreased to a value that a further increase in potential cannot compensate for the accompanied decrease in the CO_2_ concentration in the CL. Hence, CO PCD decreases with a further increase in the applied potential.

After reaching the peak CO PCD, any further reduction in CO_2_ concentration with increasing potential is primarily attributable to the Sechenov effect^23^, as both the electrochemical and homogeneous pathways for CO_2_ consumption decrease. In parallel, the ionic strength of the electrolyte steadily increases with potential due to the continuous generation of OH^−^ ions through the Hydrogen Evolution Reaction (HER). This results in a decrease in the Henry’s constant. Notably, the H_2_ PCD experiences no hindrance due to mass transport limitations, as a result of our assumption of a constant water concentration within the catalyst layer, providing a continuous supply of water through the electrolyte channel. Consequently, the HER process remains under kinetic control, showing a consistent and uninterrupted exponential growth for the entire range of potential considered (Fig. 1b).

The CO_2_ pumped through the GC inlet can undergo three different pathways; conversion into CO, consumption into ${{{{{{{{\rm{CO}}}}}}}}}_{3}^{2-}$, or leaving unreacted through the GC outlet. The first two pathways are quantified in terms of conversion and consumption efficiencies, shown in Fig. 2a. These are calculated by determining the ratio of CO molar flow rate at the GC outlet to CO_2_ molar flow rate at the GC inlet (Eq. (1)) and the ratio of ${{{{{{{{\rm{CO}}}}}}}}}_{3}^{2-}$ molar formation rate in the CL to CO_2_ molar flow rate at the GC inlet (Eq. (2)), respectively. The percentage of pumped CO_2_ leaving the GC unreacted can then be calculated using a simple carbon mass balance as shown in Eq. (3). Both conversion and consumption efficiencies follow the same trend as CO PCD with potential since the CO and ${{{{{{{{\rm{CO}}}}}}}}}_{3}^{2-}$ generation rates are directly proportional to CO PCD. Once the conversion and consumption efficiencies decrease with an increase in applied potential, a higher percentage of provided CO_2_ leaves the GC unreacted. The smallest amount of unreacted CO_2_ (90%) is predicted at −1.3 V vs RHE. This shows only a small portion of the provided CO_2_ diffuses in the GDL, of which even a smaller fraction is converted to the desired product. This inefficiency in the utilization of the provided CO_2_ occurs due to the mass transport limitation of such an electrolyzer design. However, CO_2_ mole fraction in the GC decreases continuously with increasing potential all along the electrode (Fig. 2b). This results from the dilution of the gaseous mixture in the GC with the continuously produced H_2_ in the CL from the unhindered HER. Figure 2b, c shows that the CO_2_ mole fraction decreases along the electrode for all potentials. This is due to the continuous diffusion of CO_2_ into the GDL and the diffusion of gaseous products (CO and H_2_) into the GC out of the GDL, resulting in the dilution of the gaseous mixture.

1 $${\eta }_{{{{{{{{{\rm{CO}}}}}}}}}_{2,{{{{{{{\rm{conversion}}}}}}}}}}=\frac{{\dot{{{{{{{{\rm{n}}}}}}}}}}_{{{{{{{{\rm{CO}}}}}}}},{{{{{{{\rm{GC}}}}}}}}\,{{{{{{{\rm{outlet}}}}}}}}}}{{\dot{{{{{{{{\rm{n}}}}}}}}}}_{{{{{{{{{\rm{CO}}}}}}}}}_{2},{{{{{{{\rm{GC}}}}}}}}\,{{{{{{{\rm{inlet}}}}}}}}}}$$

2 $${\eta }_{{{{{{{{{\rm{CO}}}}}}}}}_{2,{{{{{{{\rm{consumption}}}}}}}}}}=\frac{{\dot{{{{{{{{\rm{n}}}}}}}}}}_{{{{{{{{{\rm{CO}}}}}}}}}_{3}^{2-}{{{{{{{\rm{formation}}}}}}}},{{{{{{{\rm{CL}}}}}}}}}}{{\dot{{{\rm{n}}}}}_{{{{{{{{{\rm{CO}}}}}}}}}_{2},{{{{{{{\rm{GC}}}}}}}}\,{{{{{{{\rm{inlet}}}}}}}}}}$$

3 $${\eta }_{{{{{{{{{\rm{CO}}}}}}}}}_{2,{{{{{{{\rm{unreacted}}}}}}}}}}=1-{\eta }_{{{{{{{{{\rm{CO}}}}}}}}}_{2,{{{{{{{\rm{conversion}}}}}}}}}}-{\eta }_{{{{{{{{{\rm{CO}}}}}}}}}_{2,{{{{{{{\rm{consumption}}}}}}}}}}$$

**Fig. 3:**
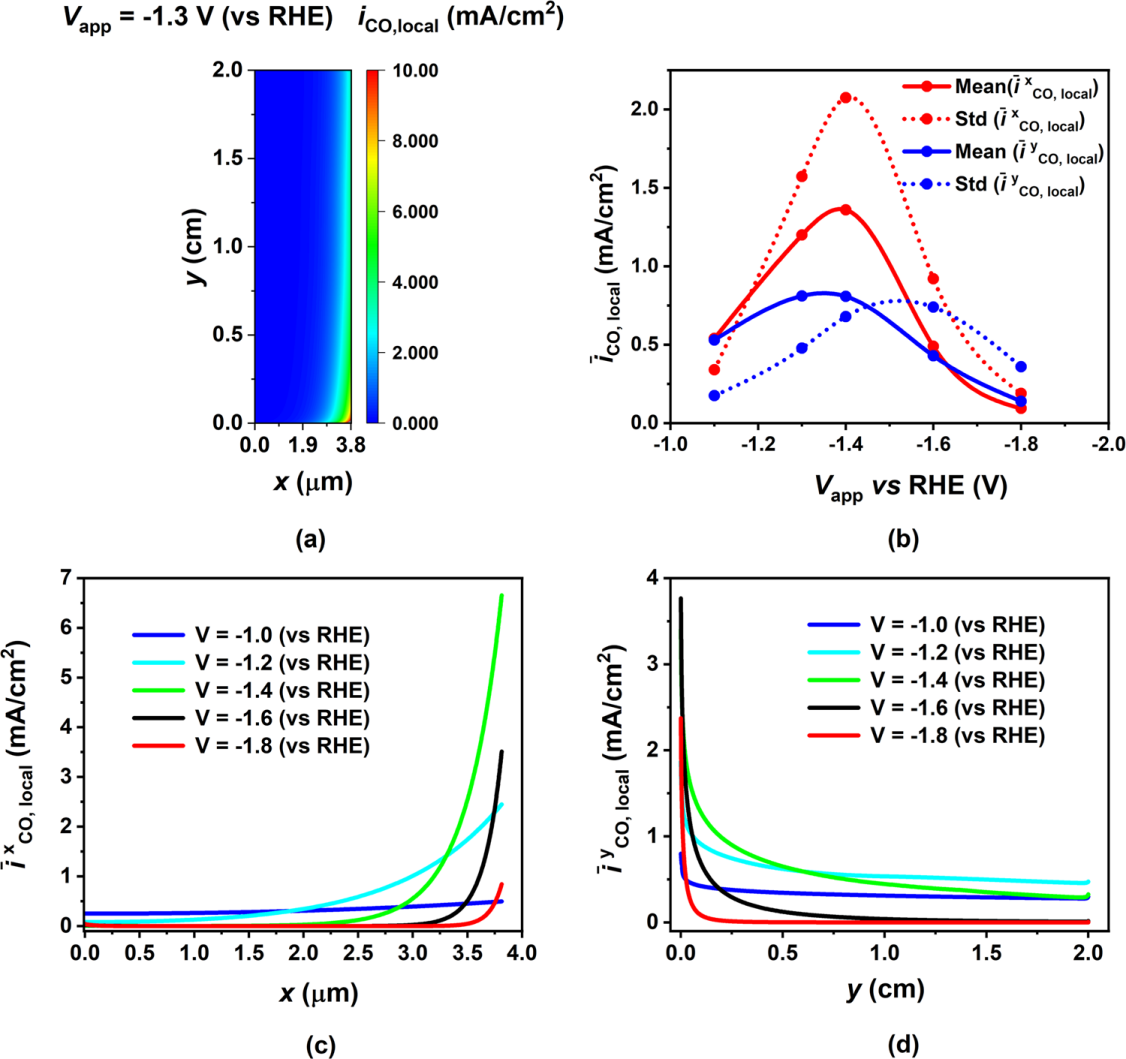
Quantifying heterogeneity in the current distribution in the catalyst layer (CL) as a function of applied cathode potential. **a** 2D contour of the local CO Partial Current Density (PCD) in the CL. **b** Mean (solid line) and standard deviation (dotted line) of the averaged local CO PCD for different applied cathode potentials. ${\overline{{i}^{x}}}_{{{\rm{CO,local}}}}$ and ${\overline{{i}^{y}}}_{{{\rm{CO,local}}}}$ denote CO PCD averaged along the y-direction (electrode length) and the x-direction (CL thickness), plotted in red and blue lines, respectively. **c** Variation of averaged local CO PCD along the CL thickness for five different applied cathode potentials between −1.0 V to −1.8 V vs RHE (blue to red curves). The local CO PCDs are averaged across the length of the electrode. **d** Variation of averaged local CO PCD along the length of the electrode in the CL for five different applied cathode potentials between −1.0 V to −1.8 V vs RHE (blue to red curves). The local CO PCDs are averaged across the thickness of the CL. The results are shown for the fully flooded CL base case the model parameters corresponding to which are given in Supplementary Tables 1 to 5 in the SI.

The 2D contour plot of the local CO PCD in Fig. 3a shows the variability in current density within the CL, at the applied potential corresponding to the peak global CO PCD. The highest current density is concentrated in the bottom-right corner of the CL, while the leftmost section exhibits minimal current production. The local current density decreases continuously as we move from the bottom to the top of the electrode and from the right (GDL-CL interface) to the left (EC-CL interface). This distribution aligns with the fact that CO_2_ concentration is highest at the GDL-CL interface, where the CO_2_ transitions from the gas phase to the aqueous phase. As we move through the thickness of the electrode, there is a gradual decrease in the CO_2_ concentration due to its hindered mass transport within the aqueous phase. We have quantified the heterogeneity in the current distribution by calculating the mean and standard deviation of averaged CO PCD, (averaged in both the x- and y-directions), as depicted in Fig. 3b. The current distribution is highly non-uniform in both directions, but it’s more pronounced in the x-direction than in the y-direction. Interestingly, we observe that the means in both directions increase with potential and then decrease, which is consistent with the global trend of CO PCD as seen in Fig. 1. Similarly, the standard deviation follows this trend, indicating that the non-uniformity in the current distribution in both directions becomes more pronounced as the total or averaged current densities increase. In Fig. 3c, d, we present the actual distribution of averaged CO PCD along both the thickness and length of the CL for various applied potentials. These figures validate the observations made in Fig. 3a regarding the current variation along both directions for a range of potentials. Furthermore, we note a sharp decline in current density beyond the point of peak global CO PCD (around −1.4 V vs RHE), indicating that significant currents are generated primarily in the initial 10% of the electrode length due to CO_2_ mass transport limitations at this stage. These observations can be attributed to localized variations in species concentrations within the CL and the gas channel, as discussed next.

**Fig. 4:**
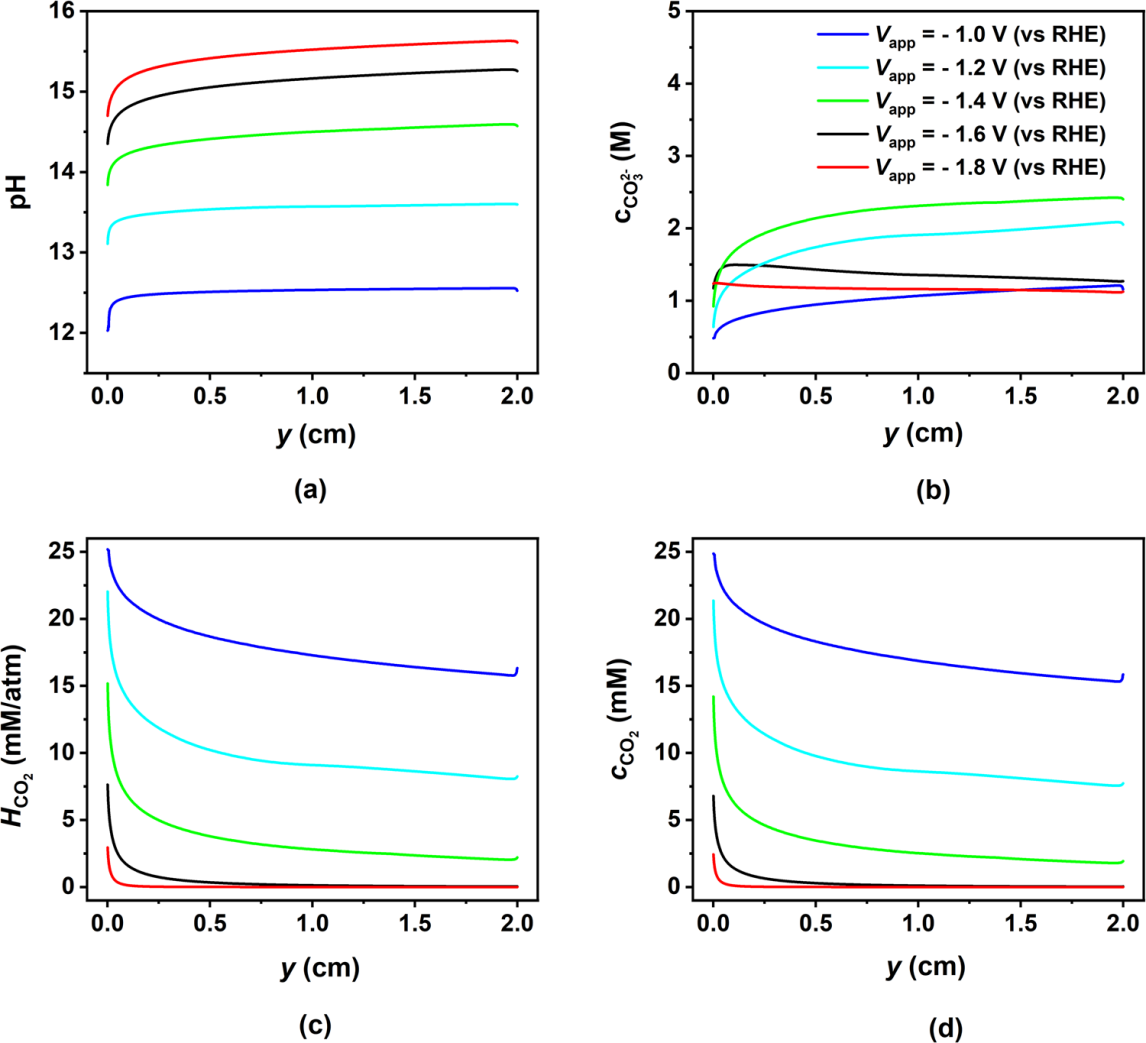
Effect of applied cathode potential on the distribution of species concentration and Henry’s constant in the catalyst layer (CL). Variation of the averaged value of **a** pH, **b** ${{{{{{{{\rm{CO}}}}}}}}}_{3}^{2-}$ concentration, **c** Henry’s constant, and **d** CO_2_ concentration along the electrode length in the CL for five different values of applied cathode potential from −1.0 V to −1.8 V vs RHE (blue to red). The quantities plotted on the y-axis are all averaged along the thickness of the CL.

Figure 4 shows the variation of CL thickness-averaged species concentration and Henry’s constant along the length of the electrode. As can be seen from Fig. 4a, the pH increases continuously along the electrode and also with potential due to the generation of OH^−^ ions from both COER and HER. The exceptionally high local pH values predicted by our model, particularly at higher applied potentials, stem from our assumption of a constant water concentration. This assumption results in H_2_O reduction to H_2_ remaining kinetically controlled across the entire potential range considered, resulting in the unimpeded production and accumulation of OH^−^ ions within the CL. Moreover, these elevated local pH levels align with previous modeling^14,15^ and experimental studies^24,25^, which have shown that local pH values can exceed 14 at high CO PCD (above 50 mA/cm^2^), even with neutral pH bulk electrolytes like 0.5 M KHCO_3_. The increase of pH along the length of the CL is a combined result of unhindered (kinetically controlled) water reduction reaction producing OH^−^ ions along with the decreasing buffering effect of flowing electrolyte. The flowing electrolyte serves as a buffer, helping to remove the OH^−^ ions generated within the CL and maintaining a consistent pH level in the CL (same as in the bulk solution). However, this buffering effect weakens as one moves further up the CL due to the increase in the boundary layer thickness of the OH^−^ ions^20^. As a result, the pH within the CL starts to rise, exacerbated by the unimpeded production of OH^−^ ions through the kinetically controlled water reduction reaction. This phenomenon leads to the observed increase in pH along the length of the CL. For potentials larger than the peak potential for CO PCD (*V*_app _≥ ≈ −1.5 V), the ${{{{{{{{\rm{CO}}}}}}}}}_{3}^{2-}$ formation reaction rate increases with increasing distance from the electrode’s bottom (due to increasing OH^−^ concentration), resulting in enhanced ${{{{{{{{\rm{CO}}}}}}}}}_{3}^{2-}$ production (Fig. 4b) and CO_2_ consumption (Fig. 4d) towards the top of the electrode. For potentials more negative than the peak potential for CO PCD, most of the upper portion of the electrode becomes depleted of CO_2_, leading to a decrease in the ${{{{{{{{\rm{CO}}}}}}}}}_{3}^{2-}$ concentration at the top. Beyond this particular potential, the decrease in CO_2_ concentration is not due to ${{{{{{{{\rm{CO}}}}}}}}}_{3}^{2-}$ formation but due to a decrease in Henry’s constant, as shown in Fig. 4c. The Henry’s constant decreases along the electrode and with potential due to the continuous increase in the ionic strength of the electrolyte in the CL, also a result of the unhindered HER. Hence, the decline in CO_2_ concentration along the CL isn’t due to electrochemical CO production but rather the formation of carbonate and decrease in the Henry’s constant at lower and higher potentials, respectively. As a result, we observe a decrease in CO PCD as we move up along the CL, as illustrated in Fig. 3d. The buildup of ${{{{{{{{\rm{CO}}}}}}}}}_{3}^{2}-$ and OH^−^ ions within the CL results in a substantial increase in the local concentration of K^+^ ions. This increase is necessary to uphold local electroneutrality. However, this heightened concentration of K^+^ ions can lead to the occurrence of salt precipitation, particularly potassium carbonate (having a solubility of 7.8M). The intricate phase transfer phenomenon, while not within the scope of this study due to its complexity and modeling challenges, has nonetheless been commonly witnessed and extensively recorded as a substantial contributor to electrode degradation and instability^26–28^.

**Fig. 5:**
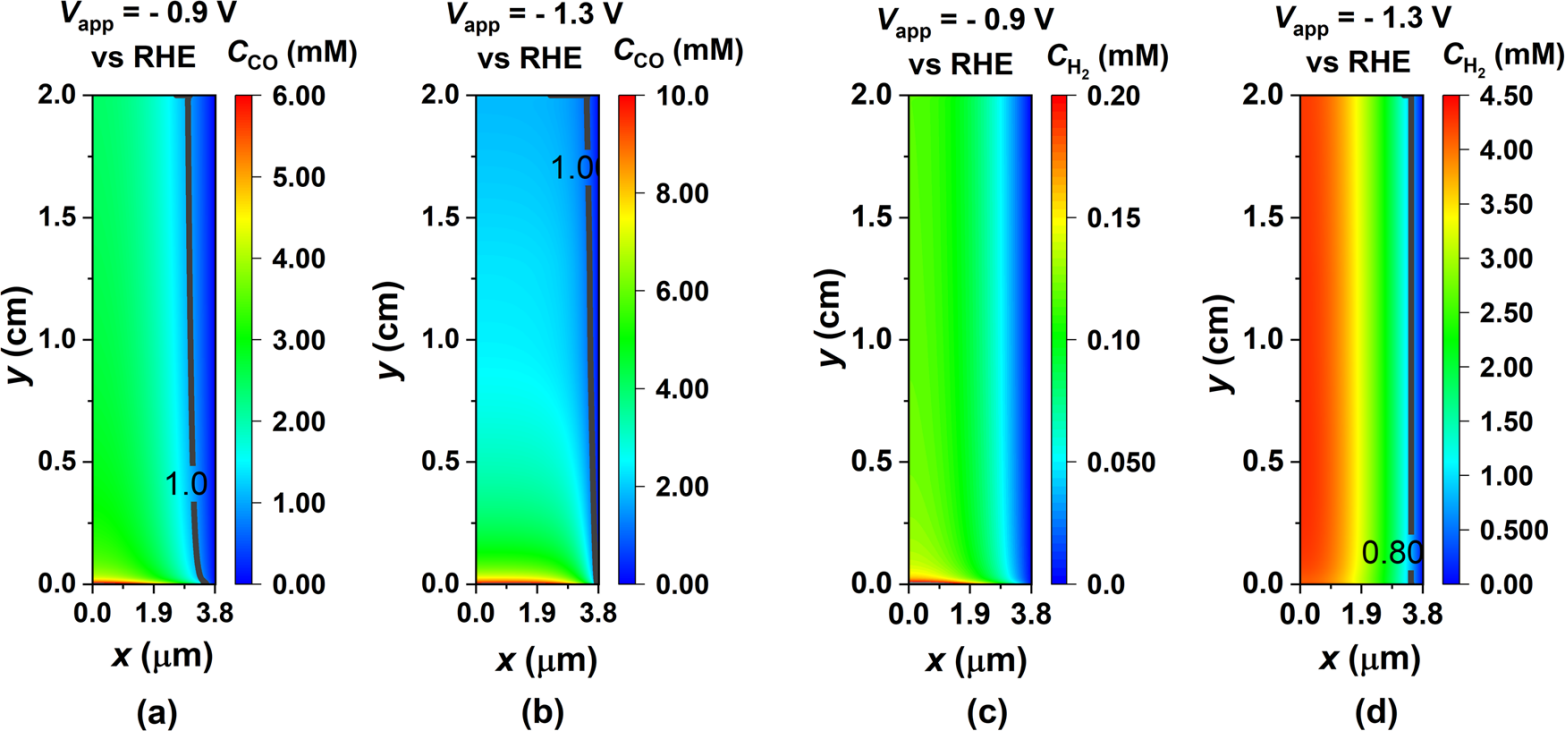
Effect of applied cathode potential on aqueous H_2_ and aqueous CO species concentration distribution in the catalyst layer (CL). 2D contour of local concentration of CO (aq.) in the CL for **a** *V*_app_= −0.9 V vs RHE and **b** *V*_app_= −1.3 V vs RHE. The contour line in (**a**) and (**b**) corresponds to the solubility limit of CO in water. 2D contour of local concentration of H_2_ (aq.) in the CL for **c** *V*_app_= −0.9 V vs RHE and **d** *V*_app_= −1.3 V vs RHE. The contour line in (**d**) corresponds to the solubility limit of H_2_ (aq.) in water.

Figure 5 displays 2D contours representing the production of electrochemical CO_2_ and H_2_O reduction products within the CL. Figure 5a, b shows contour plots depicting the distribution of CO (aq.) concentration within the CL at two different applied potentials. These plots include a contour line representing a CO concentration of 1 mM, which is the maximum solubility of CO in aqueous media. Consequently, the area to the left of this contour line is indicated as prone to bubble formation. Notably, at a higher potential (V = −1.3 V vs RHE), there is an increase in CO PCD, resulting in a higher rate of CO generation. This, in turn, leads to a larger region where bubble formation is likely to occur, as the contour line indicating the solubility limit shifts closer to the CL-GDL interface. Similarly, in Fig. 5c, d, we observe concentration contours for H_2_ within the CL at two different applied potentials. As the potential is increased to V = −1.3 V, the rate of H_2_ production rises significantly, reaching a point where H_2_ cannot remain in aqueous form throughout the CL. As a result, H_2_ bubble formation is predicted to take place on the left side of the contour line corresponding to 0.8 mM, which represents the solubility limit of H_2_ in aqueous media, as shown in 5d.

A trend apparent in the contour plots in Fig. 5 is that the lowest concentrations of electrochemical products (CO (aq.) and H_2_ (aq.)) are observed at the CL-GDL interface. This trend primarily arises from the fact that the CL-GDL interface serves as a phase transfer sink for CO (aq.) and H_2_ (aq.). Consequently, as one progresses from the EC-CL interface toward the CL-GDL interface, the concentrations of CO and H_2_ in aqueous form decrease. Previous research has noted that the formation of bubbles associated with syngas (CO+H_2_) during microfluidic CO_2_ electrolysis can negatively impact the performance of the electrolyzer^29^. In our study, we have employed modeling to identify regions within the CL that are susceptible to bubble formation. Our model incorporates CO and H_2_ within the aqueous phase, enabling us to simulate and predict regions within the CL where bubbles are likely to form. While our model can identify these regions, it does not actually simulate the bubble formation phenomenon or the subsequent two-phase flow within the CL. Nonetheless, our findings align with the bubble formation phenomenon as observed in experiments.

#### Effect of electrolyte flow rate

**Fig. 6:**
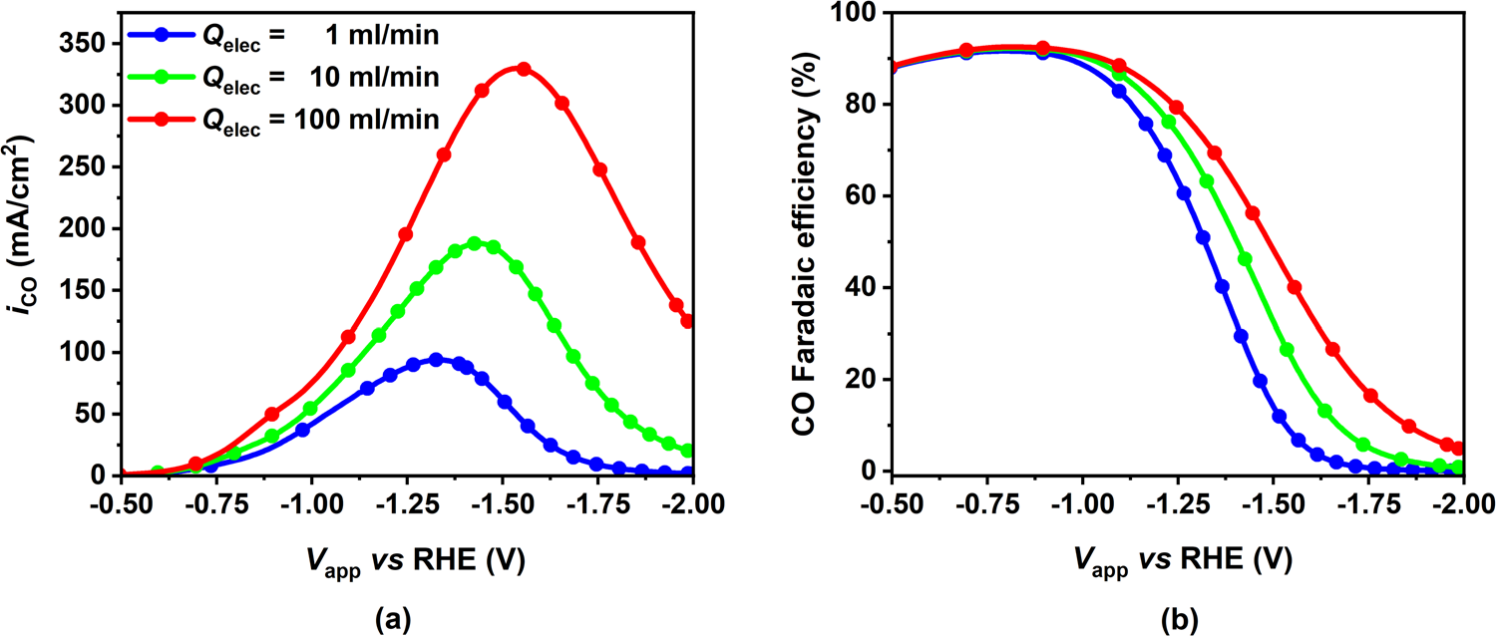
Effect of electrolyte flow rate on CO partial current density (PCD) and CO faradaic efficiency (FE) for different applied cathode potentials. **a** CO partial current density and **b** CO faradaic efficiency as a function of applied cathode potential for three different electrolyte flow rates from 1 ${{{{{{{\rm{ml}}}}}}}}/\min$ to 100 ${{{{{{{\rm{ml}}}}}}}}/\min$ (from blue to red). The model parameters correspond to the base case (fully flooded catalyst layer) mentioned in Supplementary Tables 1 to 5.

The performance of the CO_2_ electrolyzer with GDE was analyzed with respect to the applied cathode potential for different electrolyte flow rates. The CO PCD (Fig. 6a) and CO faradaic efficiency (Fig. 6b) increase as the electrolyte flow rate increases, particularly in the mass transport controlled regime (*V*_app_ ≤ ≈ −1.1 V). Moreover, the maximum CO PCD occurs at a higher applied cathode potential for higher electrolyte flow rates (−1.3 V vs RHE at 1 ml/min compared to −1.5 V vs RHE at 100 ml/min). The rise in CO FE with the electrolyte flow rate is a direct consequence of the increase in CO PCD and constant H_2_ PCD at a particular potential. For all the flow rates simulated, the trend of CO PCD with potential is comparable, with the current density initially growing exponentially (kinetically controlled regime) and then tapering off (due to mass transport limitations). H_2_ PCD, on the other hand, is solely controlled by kinetics and therefore shows an exponential growth throughout the potential range considered, resulting in a decrease in CO faradaic efficiency with larger negative applied potential.

**Fig. 7:**
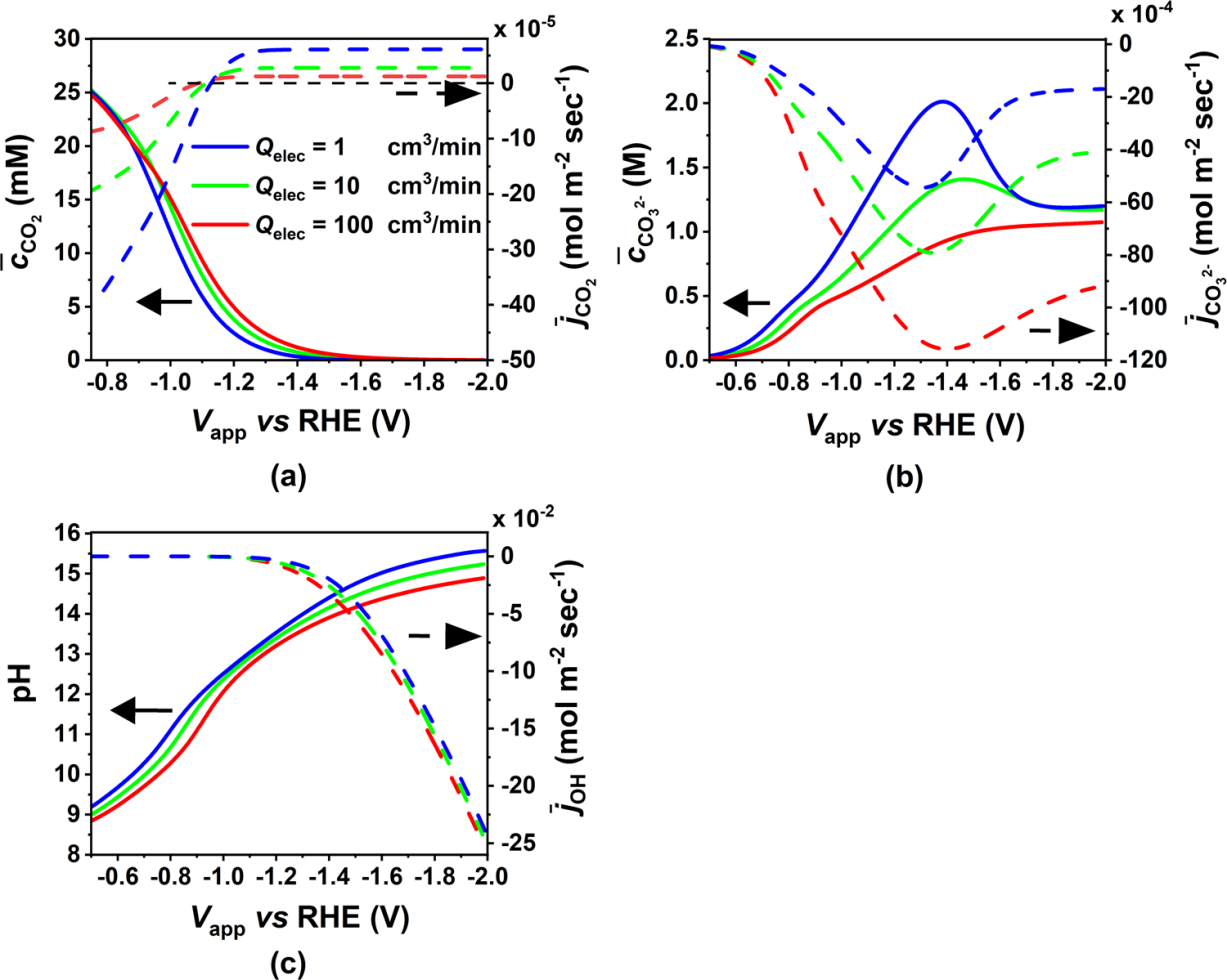
Effect of electrolyte flow rate on averaged species concentration in the catalyst layer (CL) and averaged species flux at the CL-electrolyte channel (EC) interface. Left y-axis; CL-averaged **a** CO_2_ concentration, **b** ${{{{{{{{\rm{CO}}}}}}}}}_{3}^{2-}$ concentration, **c** pH. Right y-axis; x-component of the total flux averaged along the EC/CL interface for **a** CO_2_, **b** ${{{{{{{{\rm{CO}}}}}}}}}_{3}^{2-}$, and **c** OH^−^, as a function of applied cathode potential for three different electrolyte flow rates from 1 ${{{{{{{\rm{ml}}}}}}}}/\min$ to 100 ${{{{{{{\rm{ml}}}}}}}}/\min$ (from blue to red). The solid and dotted lines denote averaged species concentration and averaged species flux, respectively. In Fig. 7a, the black dotted line indicates the zero level on the right y-axis. The model parameters correspond to the base case (fully flooded CL) mentioned in Supplementary Tables 1 to 5.

The increase in CO PCD with electrolyte flow rate can be attributed to changes in species concentration in the CL as depicted in Fig. 7. An increase in flow rate causes the concentration boundary layer of every bulk electrolyte species to become thinner, enhancing species mass transfer at the CL/EC interface. Figure 7a shows that regardless of the flow rate, the average CO_2_ concentration in the CL decreases with potential, due to a continuous decrease in the CO_2_ solubility (Sechenov effect) combined with parasitic (*i.e.* homogeneous reactions) and electrochemical consumption of CO_2_. We also observe that the CO_2_ diffusive flux becomes positive (into the CL) at higher negative potentials (above −1.15 V vs RHE) when the CO_2_ concentration in the CL falls below the bulk CO_2_ concentration in the EC. Higher flow rates enhance this diffusive flux, alleviating mass transfer limitations, resulting in higher CO_2_ concentration in the CL and thus higher CO PCD. Similarly, ${{{{{{{{\rm{CO}}}}}}}}}_{3}^{2-}$ and OH^−^ concentrations in the CL decrease with higher flow rates due to their increased diffusive fluxes out of the CL (see Fig. 7b, c). This results in decreased ionic strength of the electrolyte in the CL and subsequently a higher value of Henry’s constant, which also contributes to increased CO_2_ concentration in the CL electrolyte and hence higher CO PCD at higher flow rates. The non-monotonic nature of the ${{{{{{{{\rm{CO}}}}}}}}}_{3}^{2-}$ concentration and flux with potential results from the ${{{{{{{{\rm{CO}}}}}}}}}_{3}^{2-}$ generation rate’s direct proportionality to the CO PCD. Hence, the ${{{{{{{{\rm{CO}}}}}}}}}_{3}^{2-}$ concentration profile is similar to CO PCD, both peaking at the same potential.

#### Effect of CO_2_ gas flow rate

**Fig. 8:**
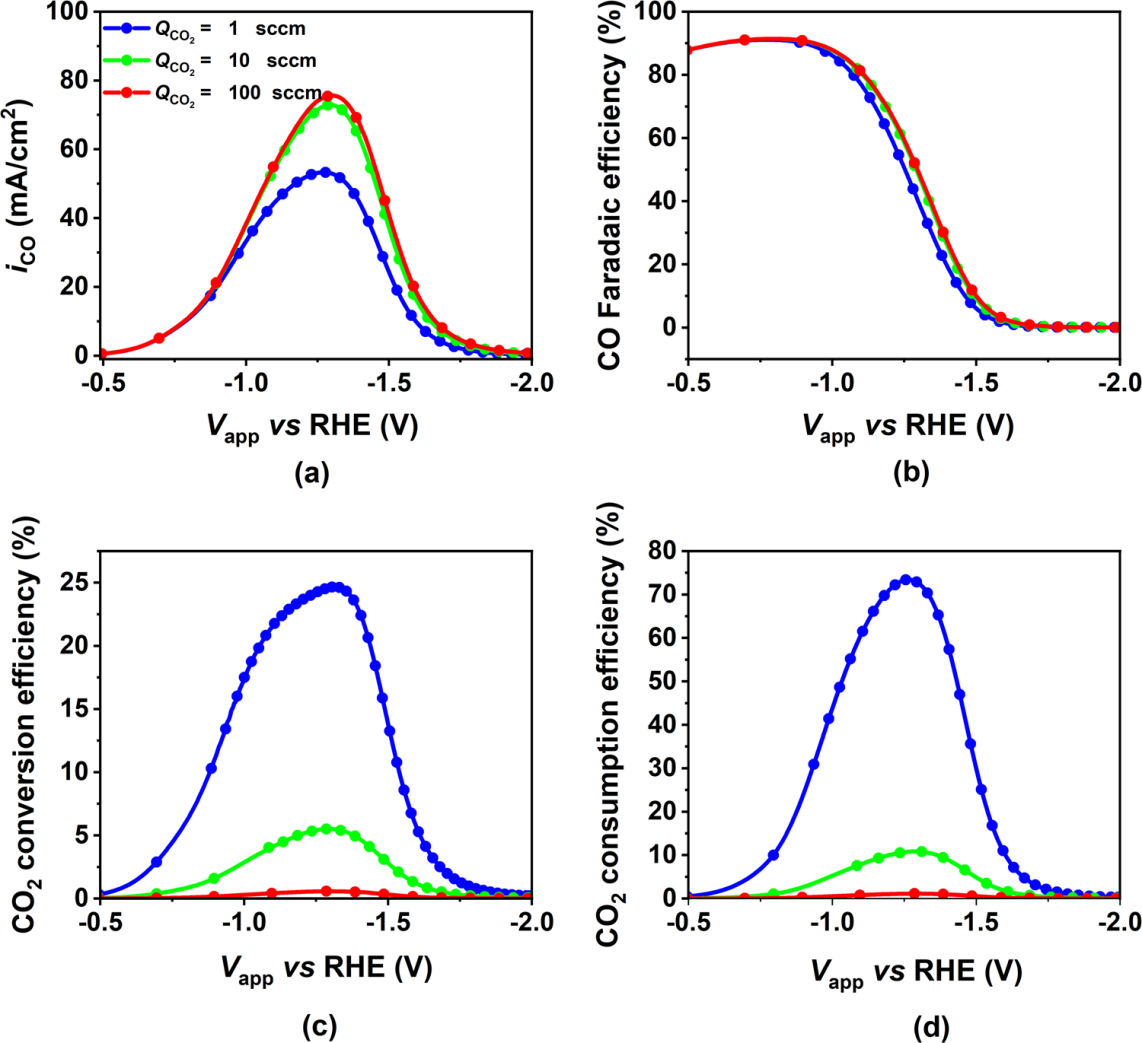
Effect of applied cathode potential on CO partial current density (PCD), CO Faradaic efficiency, CO_2_ conversion, and consumption efficiency for different CO_2_ mass flow rate. Variation of **a** CO PCD, **b** CO FE, **c** CO_2_ conversion, and **d** CO_2_ consumption, with applied cathode potential for three different values of CO_2_ gas flow rate at the GC inlet from 1 sccm to 100 sccm (from blue to red curve). The model parameters correspond to the base case (fully flooded catalyst layer) mentioned in Supplementary Tables 1 to 5.

Figure 8 shows the influence of increasing CO_2_ gas flow rates on the performance characteristics of the porous electrode. As the flow rate is increased from 1 sccm to 100 sccm, there is an increase in the CO PCD (about 10%) (Fig. 8a) and consequently in the FE (Fig. 8b). This increase is only noticeable once the kinetically controlled regime ends and the mass transport regime begins (*V*_app_ ≤ −1.1 V vs RHE). For all the flow rates simulated, the CO_2_ conversion and consumption efficiency curves have a shape similar to the CO PCD curve. This results from the direct relation between CO PCD and the rates of CO and ${{{{{{{{\rm{CO}}}}}}}}}_{3}^{2-}$ generation, which correspond to the conversion and consumption efficiencies, respectively. Nonetheless, these efficiency values drop drastically with an increase in flow rate, implying a corresponding rise in the fraction of unreacted CO_2_ leaving the GC. The unreacted CO_2_ leaving the device increases from 5% to 97% at ~ −1.4 V vs RHE when increasing the CO_2_ gas flow rate from 1 to 100 sccm, while the CO PCD increased from 50 to 75 mA/cm^2^.

The increase in CO PCD can be explained in terms of an increase in CO_2_ concentration in the CL with the flow rate (see Supplementary Fig. 2a in the SI). The increase in CO_2_ concentration is not substantial, which explains the lower increase in peak CO PCD (around 50%) compared to the increase in the electrolyte flow rate (around 400%) when increasing the flow rate by a factor of 100. This increase in CO_2_ concentration can be explained in terms of an increase in CO_2_ mass flow rate at the GDL/GC interface (Supplementary Fig. 2b (left y-axis)). However, while higher CO_2_ gas flow rates alleviate mass transport limitations for COER, the increase in CO PCD is not proportional to the increase in CO_2_ gas flow rate. Consequently, CO_2_ conversion efficiency decreases. This hypothesis is supported by Supplementary Fig. 2b (right y-axis), which demonstrates that as the CO_2_ gas flow rate increases, the fraction of CO_2_ mass flow rate entering the GDL relative to that entering the GC inlet decreases. As a result, only a small fraction of the additional CO_2_ provided at the larger flow rate is utilized to increase the rate of COER, while the rest exits from the GC outlet unreacted, decreasing both the CO_2_ conversion and consumption efficiency.

### Effect of CL properties

Here, we aim to provide guidance on how to engineer CL properties to improve mass transport limitations. To achieve this, we first examine how the CL’s performance changes when its material composition is altered. Subsequently, we delve into the separate analysis of two key CL properties: CL porosity and CL anisotropy in diffusion. We explore how these properties can be adjusted to limit mass transport limitations and optimize the CL’s performance in terms of CO PCD and CO FE.

Supplementary Fig. 3 in the SI, presents a performance comparison between two realistic CLs fabricated with Cu and Ag. The properties of these CLs have been calculated based on direct pore-level simulations on the exact CL structure obtained by FIB-SEM nano-tomography^21^ and are implemented in our model. We assume that the two CLs represent typical CL structures that are assumed selective to CO. The aim is to understand if the variation in performance stems from the inherent disparities in effective transport and geometrical properties of specific CLs. In our model the electrode kinetic parameters remain the same for both electrode materials; the distinction is drawn based on differences in their transport and geometrical properties. As observed in Supplementary Fig. 3a, b, CLs with structures with effective transport and morphological properties resembling our realistic Ag sample demonstrate superior performance in terms of both CO PCD and CO FE compared to CL structures, who’s transport and morphological characteristics resemble our realistic Cu sample. The CO PCD and CO FE curves exhibit a similar pattern to our base case results in Fig. 1, initially increasing with potential and then decreasing, for reasons explained in Fig. 1. Given the coupled nature of the process, it is challenging to pinpoint the exact factors that give the Ag-resembling CL the edge over the Cu-resembling CL. The changes stem from the combined influence of six transport and morphological parameters that differ between the two CLs, as outlined in Supplementary Table 6 in the SI. The purpose of presenting these results is to underscore the significant impact that the CLs morphology and effective transport properties can have on overall performance. In the subsequent sections, we narrow down our focus to two of these six distinct parameters.

#### Effect of heterogeneity in CL porosity

**Fig. 9:**
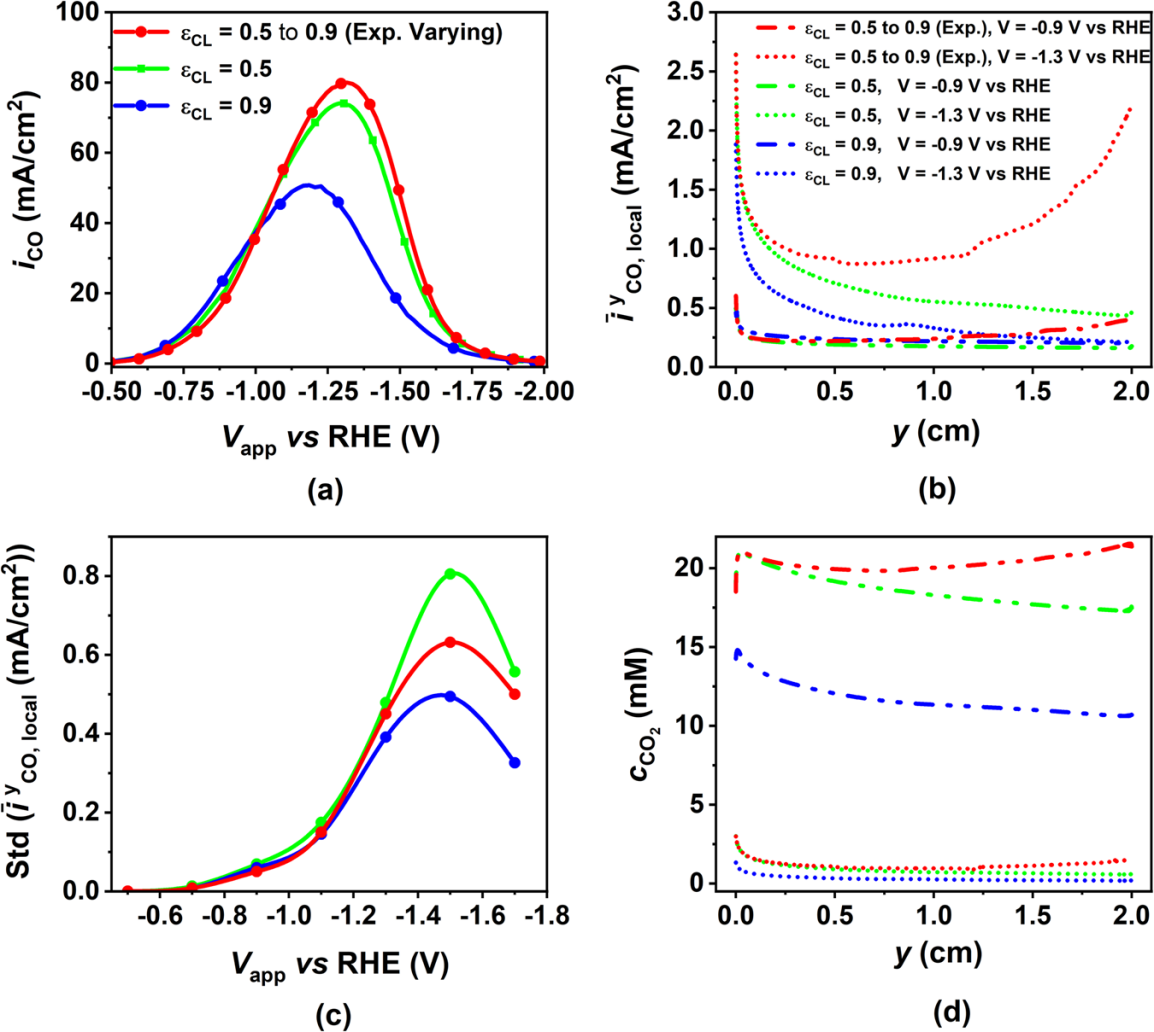
Effect of applied cathode potential on CO partial current density (PCD) along with its distribution in the catalyst layer (CL) and averaged CO_2_ concentration in the CL for different CL porosities. **a** CO PCD as a function of applied cathode potential for different CL porosities. Variation of **b** averaged local CO PCD and **d** CO_2_ along the electrode length for three different cases of CL porosity at two different values of potential. The dash-dotted and dotted lines are for applied cathode potentials V = −0.9 V vs RHE and V = −1.3 V vs RHE, respectively. **c** The standard deviation of the averaged CO PCD as a function of applied potential. Green and blue curves correspond to constant porosity values while red corresponds to exponentially varying porosity starting from 0.5 at the bottom of the CL to 0.9 at the top.

Here, we show the effect of varying the CL porosity on electrode performance. The porosity of the CL indirectly influences other morphological and transport parameters. Specifically, it impacts parameters such as the specific surface area (${a}_{v}=3\frac{(1-{\epsilon }_{CL})}{{r}_{np}}$), effective electronic conductivity, and effective diffusivity, all through the application of the Bruggeman correlation (${\sigma }_{{{{\rm{cor}}}}}={(1-{\epsilon }_{CL})}^{1.5}$ and ${D}_{{{\rm{cor}}}}={\epsilon }_{CL}^{1.5}$). Through these relationships, we can see that an increase in CL porosity results in a decrease in both *a*_*v*_ and *σ*_cor_, while it leads to an increase in *D*_cor_. Thus, porosity has a dual impact on both transport and electrode kinetic phenomena within the CL and these effects run in opposite directions. An increase in porosity enhances the transport phenomenon due to the increase in *D*_cor_ while it negatively impacts the kinetics as both *a*_*v*_ and *σ*_cor_ decrease. Figure 9a shows CO PCD as a function of applied cathode potential for three different porosity distributions in the CL. While the green and the blue curves correspond to constant porosity distribution, the red curve assumes that the CL porosity varies exponentially from 0.5 at the bottom of the electrode to 0.9 at the top. There is a clear increase in the maximum CO PCD with a decrease in the porosity (50% increase when going from 0.9 to 0.5). However, changing a homogeneously porous CL to a heterogenous one, there is only a slight increase in the maximum CO PCD (8% only). As previously discussed, varying CL porosity has the opposite impact on electrode kinetics and electrode transport phenomenon. From Fig. 9a, it is evident that the reduction in *a*_*v*_ and *σ*_cor_ with increase in porosity dominate the increase in transport of species through the increase in *D*_cor_.

The introduction of CL porosity heterogeneity improves CL performance by reducing current distribution variability in the y-direction (Fig. 9b). Analyzing the standard deviation of current distribution in the y-direction (Fig. 9c), we observe a lower standard deviation for heterogeneous porosity compared to homogeneous porosity (0.5). However, the lowest standard deviation remains in the homogeneous porosity case (0.9), mainly due to its low overall CO PCD. Enhanced mass transfer in the y-direction results in more uniform CO_2_ concentration distribution along the CL length (Fig. 9d) within the heterogeneous case, ensuring uniform current generation. Increased porosity at the electrode’s upper part acts as a buffer, aiding in the removal of detrimental electrochemical reaction products like hydroxide and carbonate ions, thus maintaining a higher, uniform CO_2_ concentration throughout the CL. In summary, introducing heterogeneity in CL porosity mitigates species distribution variability, thereby overcoming mass transport limitations and improving overall CO PCD.

#### Effect of anisotropy in CL’s effective diffusivity

**Fig. 10:**
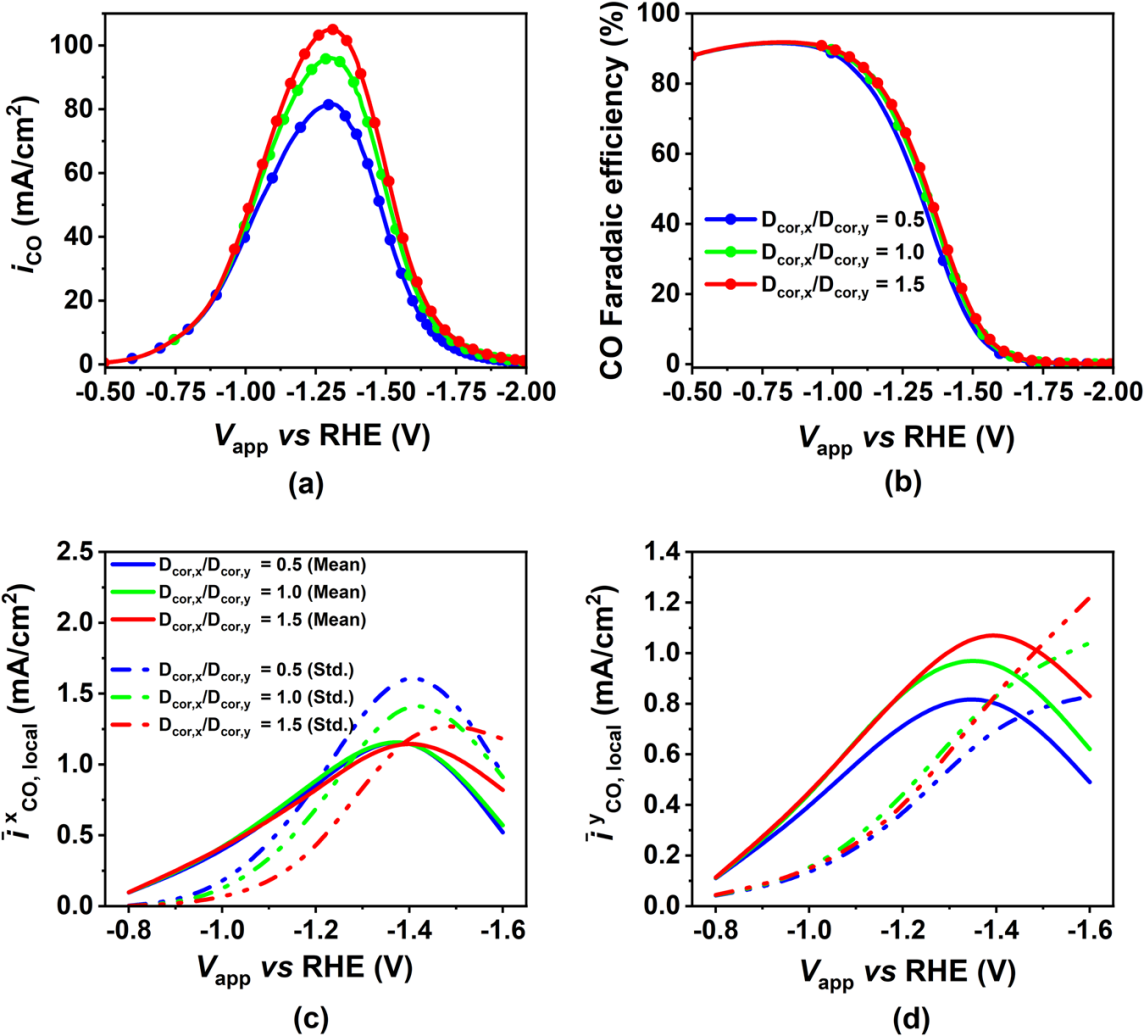
Effect of applied cathode potential on CO partial current density (PCD), CO faradaic efficiency (FE), and heterogeneity in CO PCD distribution in the x- and y-directions in the catalyst layer (CL) for varying levels of anisotropy in CL diffusivity correction. **a** CO PCD and **b** CO FE as a function of applied cathode potential for different ratios of diffusivity correction in the x-direction to the diffusivity correction in the y-direction (D_cor,y_ is fixed as 1) in the CL. Mean and standard deviation of **c** y-averaged local CO PCD **d** and x-averaged local CO PCD as a function of applied cathode potential for different cases of CL anisotropy.

The porosity of the CL impacts both the kinetics and transport processes within it. To isolate these influences on electrode performance, we aim to tweak a specific CL property that exclusively affects transport processes within the CL. We investigate the anisotropic nature of CL diffusivity correction, a CL characteristic evident in the data presented in Supplementary Table 6. Through our homogenized volume-average model, we investigate how manipulating this CL characteristic can improve electrode performance. For this, we simulate three different ratios of CL diffusivity correction, by keeping the CL diffusivity correction in the y-direction as constant (=1) and varying the diffusivity correction in the x-direction. In Fig. 10a, b, we see as the ratio of diffusivity correction increases, both CO PCD and CO FE show improved electrode performance. Moving on to Fig. 10c, d, we assess the current distribution heterogeneity in the CL along the x- and y-directions. We achieve this by plotting the mean and standard deviation of the current distribution in these directions as a function of applied cathode potential. As expected, the mean of the current distribution in both directions aligns with the trend seen in CO PCD (Fig. 10a), with the highest mean occurring for the highest diffusivity correction ratio. However, the more interesting observation pertains to the variation of standard deviation with diffusivity correction. In the x-direction, the standard deviation decreases as the x-direction diffusivity correction increases. This suggests a more uniform current distribution along the x-direction, extending current generation from the CL-GDL interface towards the EC-GDL interface, effectively utilizing more of the CL for current generation. This can be explained in terms of improvement in the mass transport of species (both reactants and products) on increasing the diffusivity correction in the x-direction. Conversely, in the y-direction, the standard deviation does not decrease with an increase in the diffusivity correction ratio. This outcome was expected since we varied the diffusivity correction ratio by keeping the y-direction constant and adjusting the x-direction. Consequently, the heterogeneity in the y-direction increases due to the overall increase in CO PCD with higher diffusivity correction. In summary, the augmentation of diffusivity correction in the x-direction enhances mass transport efficiency. This improvement effectively surmounts mass transport limitations, resulting in a more consistent current generation across the CL and an overall boost in CO PCD and CO FE especially at higher potentials. We note that it is likely that most CLs do have an anisotropic nature^21^ and consequently all previous models neglecting the anisotropy of the CL do not accurately represent CLs.

## Conclusions

A 2D volume-averaged model of a microfluidic CO_2_ electrolyzer with a gas diffusion cathode was developed and compared against experimental data. This model accounts for the lateral flow (perpendicular to current flow) and includes modeling of electrochemical products (CO and H_2_) in both liquid and gas phases. Two scenarios were used to model the liquid phase distribution in the catalyst layer: ideally wetted and fully flooded. Comparatively, the fully flooded scenario better matched experimental data and was chosen as the base case.

The validated model was employed to investigate the impact of operational conditions and CL properties on performance. The model revealed two distinct electrochemical regimes. Initially, at lower negative potentials (roughly up to −1.5 V vs RHE), there exists a kinetically controlled regime where CO production exponentially increases with applied potential. Subsequently, a mass transport controlled regime is observed, characterized by peak CO PCD. Further potential increase leads to a decline in CO PCD. This mass transport limitation arises from the continuous decrease in aqueous CO_2_ concentration within the catalyst layer due to the formation of ${{{{{{{{\rm{CO}}}}}}}}}_{3}^{2-}$ ions. In addition, ongoing generation of ${{{{{{{{\rm{CO}}}}}}}}}_{3}^{2-}$ and OH^−^ ions raises the electrolyte’s ionic strength, reducing CO_2_ solubility in the aqueous electrolyte within the catalyst layer, further hindering CO_2_ transport. In contrast, H_2_ production remains nearly unaffected by mass transport limitations, displaying continuous current density growth.

To mitigate CO_2_ mass transport limitations at high potentials, increasing the electrolyte or CO_2_ gas flow rate proves effective. A substantial (400%) rise in peak CO partial current density (PCD) was achieved by increasing the electrolyte flow rate from 1 ml/min to 100 ml/min. Increasing the CO_2_ gas flow rate from 1 sccm to 100 sccm resulted in a 50% PCD increase. Increasing the electrolyte flow rate improves CO_2_ diffusion from the electrolyte channel to the catalyst layer. This replenishes CO_2_ concentration in the CL and helps remove accumulated OH^−^ and ${{{{{{{{\rm{CO}}}}}}}}}_{3}^{2-}$ ions. Consequently, it lowers the CL pH and provides buffering. Increasing the CO_2_ mass flow rate at the gas channel inlet leads to improved CO_2_ transport within the GDL. However, this enhancement comes at the cost of significantly reduced conversion efficiency. The majority of the increased CO_2_ flow exits the GC without undergoing reactions within the GDL, resulting in reduced overall conversion efficiency.

We analyzed the local distribution of CO and H_2_ (aq.) concentration in the CL, revealing that at higher potentials, these concentrations exceed their solubility limits, which leads to bubble formation and electrode instability, as has been observed experimentally. The model also provides insights into the heterogeneity of current generation within the catalyst layer in both lateral (along the CL) and longitudinal (across the CL) directions. In the fully flooded CL scenario, CO PCD exhibited significant heterogeneity, with most current generated near the CL-GDL interface. This resulted in substantial unused CL area due to CO_2_ (aq.) mass transport limitations. To address this, we introduced heterogeneity in CL porosity, varying exponentially from 0.5 to 0.9, increasing from the bottom to the top of the CL. The more porous upper portion effectively buffered species concentrations, promoting a more uniform CO_2_ distribution along the CL length and increasing overall CO PCD. We investigated anisotropy in porous CL diffusivity as another material property aspect to help overcome the mass transport limitation. By increasing diffusivity correction in the x-direction compared to the y-direction, CO_2_ mass transport limitation was alleviated within the CL. This resulted in a more even CO_2_ distribution and higher CO PCD, effectively utilizing more of the CL’s catalyst surface area.

This study identifies limitations in the performance of aqueous-electrolyte-based GDEs for CO_2_ electrolysis. It highlights the challenges posed through the coupling between CO_2_ electroreduction and CO_2_ consumption to form ${{{{{{{{\rm{CO}}}}}}}}}_{3}^{2-}$ through hydroxide ions, as well as the accumulation of hydroxide and carbonate ions in the electrolyte. Mass transport limitations arising from these conditions introduce heterogeneities in the current distribution within the CL, leading to underutilization of the available CL area for current generation. To address these challenges, one proposed solution involves incorporating porosity variation in the CL, gradually increasing from the bottom to the top of the electrode. In addition, designing anisotropic catalyst layers with enhanced through-plane diffusivity has been shown to be effective in overcoming these limitations. These strategies have demonstrated effectiveness in mitigating heterogeneity within the CL and consequently improving the performance of the aqueous CO_2_ reduction device, particularly in terms of CO PCD.

## Methods

### Physical model

**Fig. 11:**
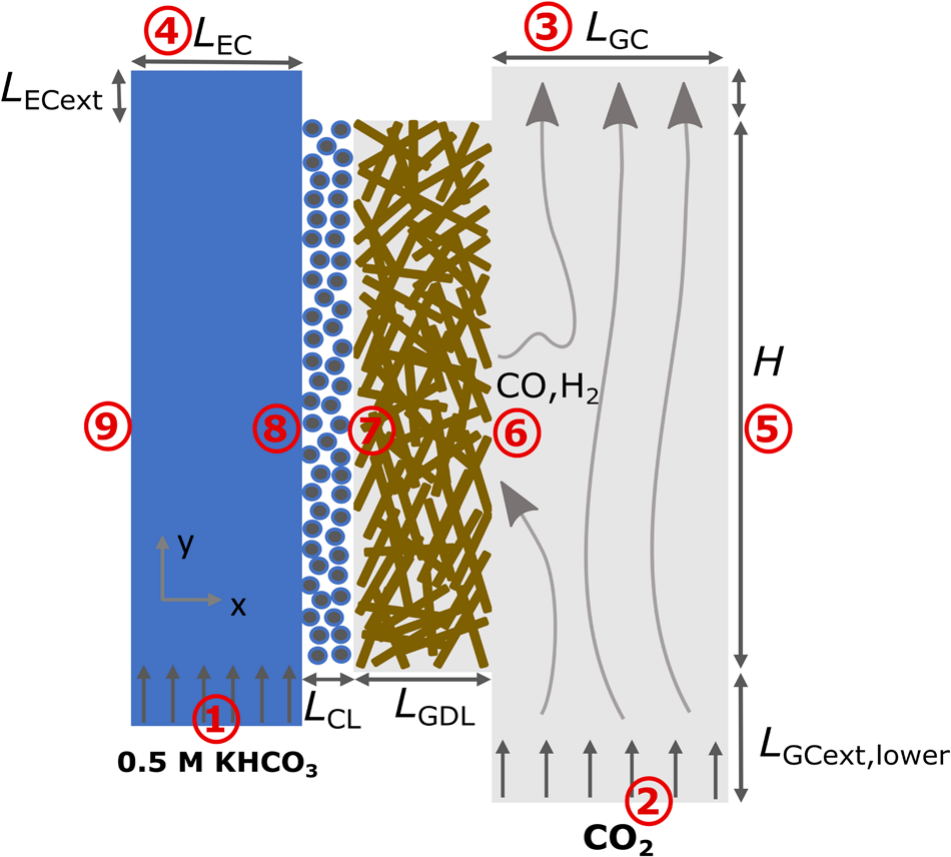
Schematic representation of the 2D modeling domain of an aqueous electrolyte-based gas diffusion electrode (GDE), showing different components of the modeled half-cell. The schematic shows the catalyst layer (CL) and the gas-diffusion layer (GDL) sandwiched between the electrolyte channel (EC) and gas channel (GC). GC and EC domains have been extended on both inlet and outlet sides for numerical convenience. Subscript ECext and GCext, lower denote extension lengths for EC and the inlet of the GC. Boundary 1 and 2 represent inlets to EC and GC, respectively, whereas 4 and 3 represent their outlets. Boundaries 6 and 7 is the interface of the GDL with GC and CL, respectively. Boundary 8 is the EC/CL interface. Boundaries 5 and 9 are the outermost boundaries of the simulated domain. Boundary 9 represents EC interface with the ion-exchange membrane connecting the cathode half cell with anode half cell. The boundary conditions for all the labeled boundaries are mentioned in Table 1.

Figure 11 shows a schematic representation of the 2D modeling domain of the developed volume-averaged model of a GDE integrated with electrolyte and gas-flow channels. It includes four modeling domains representing the electrolyte flow channel (EC), catalyst layer (CL), gas diffusion layer (GDL), and gas-flow channel (GC). The model boundaries are labeled in the schematic and detailed in Table 1. The model parameters corresponding to the geometry of the modeling domain are summarized in Supplementary Table 1. Aqueous 0.5 M potassium bicarbonate solution and 100% gas phase CO_2_ at atmospheric pressure are fed through the electrolyte and gas flow channels, respectively. Some of the CO_2_ pumped in through the GC diffuses into the GDL and the rest leaves the GC unreacted.

**Table 1:** Boundary conditions for species, potential, and flow as per numbering in Fig. 11

Boundary	Species	Potential	Flow
Boundary 1	Bulk Concentration	Insulation	Uniform
	0.5 M KHCO_3_		electrolyte velocity
Boundary 2	Constant mass flow	Insulation	Uniform
	rate of CO_2_		gas velocity
Boundary 3	Outflow boundary	Insulation	Atmospheric
	(zero-diffusive flux)		pressure
Boundary 4	Outflow boundary	Insulation	Atmospheric
	(zero-diffusive flux)		pressure
Boundary 5	Impermeable wall	NA	Zero
	(zero gaseous flux)		gas velocity
Boundary 6	Continuity of gaseous	Applied electrode	Continuity
	species concentration	potential (vs SHE)	of gas-phase velocity
Boundary 7 (I.W)	No flux of	Zero electrolyte	Continuity
	aqueous species	current	of gas-phase velocity
Boundary 7 (F.F)	Phase transfer reactions	Zero electrolyte	Zero gas velocity
	(Eq. (33) and Eq. (34))	current	
Boundary 8 (I.W)	Continuity of aqueous	Zero electrolyte	Zero electrolyte
	species concentration	potential (vs SHE)	and gas velocity
Boundary 8 (F.F)	Continuity of aqueous	Zero electrolyte	Zero
	species concentration	potential (vs SHE)	electrolyte velocity
Boundary 9	${n}_{{{{{{{{{\rm{HCO}}}}}}}}}_{3}^{-},x}=\frac{{i}_{{{{{{{{\rm{l}}}}}}}}}}{F}$	*i*_l_ = avg. (*i*_s_)	Zero
		(*i*_*s*_ at GDL-GC interface)	electrolyte velocity

**Fig. 12:**
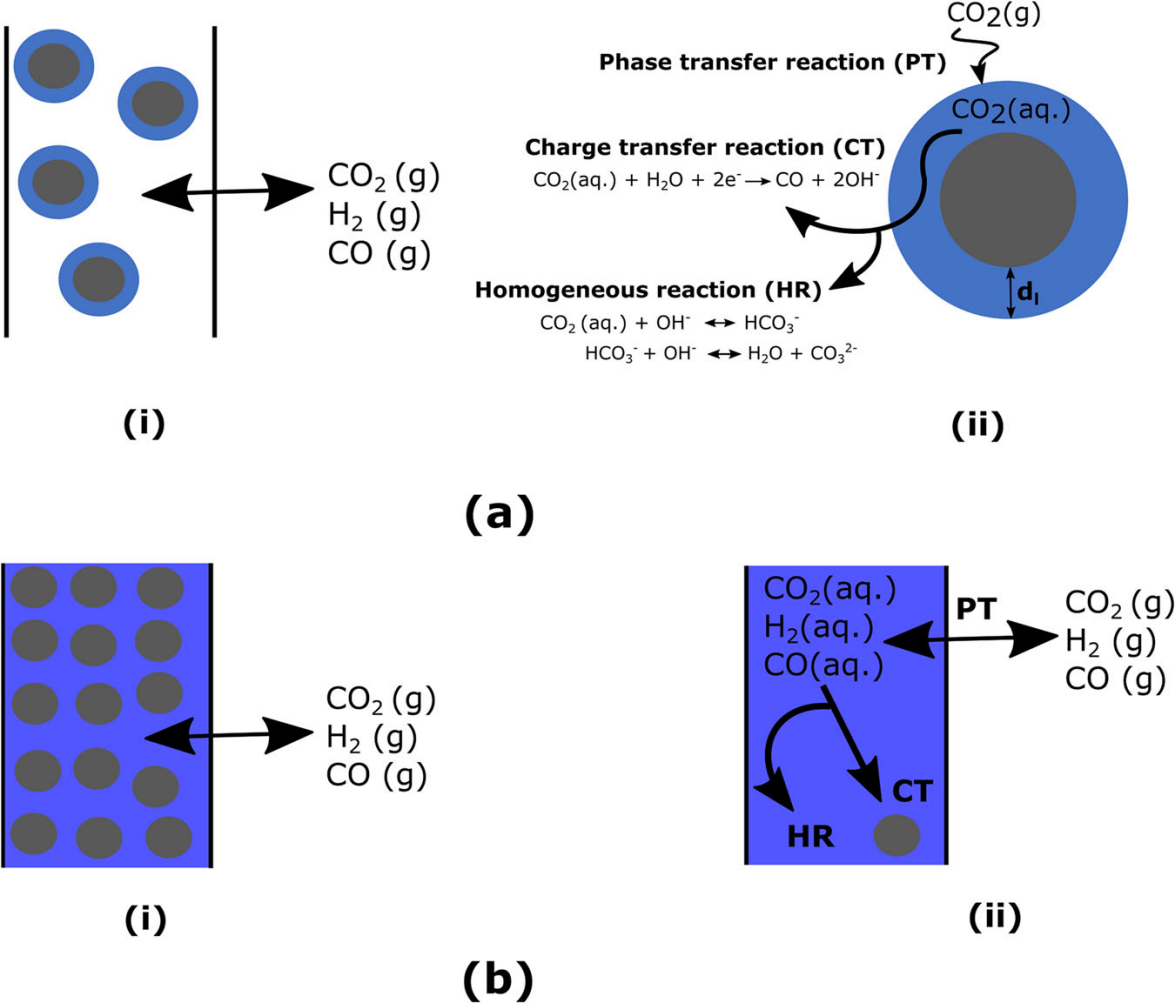
Ideally wetted and fully flooded representations of the catalyst layer (CL). **a** Ideally wetted representation of the CL, showing (i) a portion of the CL containing ideally wetted catalyst nanoparticles and (ii) a zoomed-in view of a silver catalyst nano-particle from the CL, covered by a thin aqueous electrolyte film. CO_2_ dissolved in this electrolyte film can either get converted to CO or get consumed to form ${{{{{{{{\rm{CO}}}}}}}}}_{3}^{2-}$, through the reactions shown. **b** Fully flooded representation of the CL, showing (i) a portion of the CL containing catalyst nanoparticle fully flooded with electrolyte and (ii) a schematic showing the phase transfer reactions happening at the CL-GDL boundary, while the homogeneous (HR) and charge transfer (CT) reactions occur in the bulk of CL.

Figure 12 illustrates two different strategies to model the phase distribution in the CL corresponding to two extreme saturation conditions. Figure 12a (i) shows the best-case scenario of CL wetting referred to as the ideally wetted (I.W) case, while Fig. 12b (i) shows the worst-case scenario where all the catalyst nanoparticles are fully submerged in electrolyte referred to as the fully flooded (F.F) case. For the ideally wetted case, the catalyst nanoparticle is assumed to be covered with a uniform 10-nm-thick electrolyte film as shown in Fig. 12a (ii). This thickness corresponds to the CL saturation at zero capillary pressure and a saturation (*S*) value of 0.64 which represents an average electrolyte volume fraction occupation in the pores of the CL^15^. The schematic also shows the different reactions that take place in the CL. The gaseous CO_2_ transported in the CL dissolves in the aqueous electrolyte, after which it can either get consumed to form ${{{{{{{{\rm{CO}}}}}}}}}_{3}^{2-}$ through the CO_2_ acid/base homogeneous reactions or get transported to the catalyst surface to get electrochemically reduced to CO. For the F.F CL case, CL is devoid of any gaseous species. All the gaseous species phase transforms to aqueous phase at the CL-GDL interface as shown in Fig. 12b (ii). GDL is considered hydrophobic (no aqueous species present)^15^ for both I.W and F.F CL cases. Usually in the experimental setup of such CO_2_ electrolyzer systems, the CL is prepared by mixing catalyst nanoparticles and ionomer (usually Nafion) together and coating it on the GDL. Ideally, this process results in the formation of two distinct interfaces within the CL: one between the catalyst nanoparticles and the ionomer, and one between the ionomer and the aqueous electrolyte. In this study, we have chosen to omit the ionomer, focusing solely on the interface between the catalyst nanoparticles and the aqueous electrolyte. This is done to reduce the complexity of the already intricate multi-physical phenomena in the CL. Future models should include the ionomer to assess its effect on the performance.

### Boundary conditions

At the inlet of the EC, bulk concentrations of different electrolytic species, corresponding to a 0.5 M KHCO_3_ solution, are specified. The mole fraction of gaseous CO_2_ is specified as one at the inlet of the GC. At the outlet of GC and EC, we assume zero-diffusive flux. Uniform electrolyte and gas flow velocities are specified at the EC and GC inlet, respectively, and atmospheric pressure is specified as the flow boundary condition at the outlet of EC and GC. Cathode potential (vs SHE) is applied at the GDL/GC interface, while zero electrolyte potential (vs SHE) is applied at EC/CL interface as a reference. The boundaries denoted as 7 and 8 in Fig. 11 are the points of distinction between the two CL modeling cases (I.W and F.F), and these differences are further detailed in Table 1. In the I.W case, the gas phase velocity is set to zero at boundary 8, while in the F.F case, boundary 7 serves as the point where the gas phase velocity becomes zero (as gas cannot enter the CL). In the I.W case, the flux of aqueous and gaseous species are set as zero at boundary 7 and 8, respectively. In the F.F case, phase transfer reactions occur at boundary 7, and these reactions are implemented through Eq. (33) and Eq. (34).

Boundary 9 is considered to be a membrane boundary for a ${{{{{{{{\rm{HCO}}}}}}}}}_{3}^{-}$ specific anion-exchange membrane. This boundary condition is implemented by allocating the transport of total current exclusively to ${{{{{{{{\rm{HCO}}}}}}}}}_{3}^{-}$ ions through that boundary, while the flux of all other ions is constrained to zero at that particular boundary. The type of ions that transport the charge across the membrane can change depending on the current density of the entire cell. However, we have made the simplifying assumption that only bicarbonate ions are responsible for conducting all the current under all studied operating conditions. A comprehensive electrolyzer model for the entire cell is needed to account for the more realistic situation. Base case operating parameters corresponding to all the boundary conditions are mentioned in Supplementary Table 3. These parameters correspond to the conditions used in the experimental study by Verma et al.^13^.

### Mathematical model

The multi-physics model utilizes macroscopic governing equations for the conservation of mass, momentum, and charge, along with specified boundary conditions to solve for the local species concentrations, electrode and electrolyte potentials. A brief description of these governing transport equations along with their source terms arising from different reactions occurring in the CL is provided below. The model parameters corresponding to different reactions are given in Table Supplementary Table 2 of the SI.

#### Transport equations

We review the transport equations for mass, momentum, and charge. The equations in the GDE are applicable to both 1D and 2D GDE models, while those in the electrolyte and the gas channels are applicable only for the 2D GDE model. The schematic (Supplementary Note 1 and Supplementary Fig. 1) and the boundary conditions (Supplementary Note 2) for the 1D GDE model are provided in Supplementary Methods 1 of the SI.

##### Momentum conservation equations

The flow field inside the electrolyte flow channel is solved using laminar incompressible form of the Navier-Stokes equations which are composed of the mass continuity and the momentum balance equation given by,

4 $$\nabla \cdot \overrightarrow{{{{{{{{{\rm{u}}}}}}}}}_{{{{{{{{\rm{e}}}}}}}}}}=0$$

5 $${\rho }_{{{{{{{{\rm{e}}}}}}}}}\left(\overrightarrow{{{{{{{{{\rm{u}}}}}}}}}_{{{{{{{{\rm{e}}}}}}}}}}\cdot \nabla \right)\overrightarrow{{{{{{{{{\rm{u}}}}}}}}}_{{{{{{{{\rm{e}}}}}}}}}}=\nabla {{{{{{{\rm{p}}}}}}}}+{\mu }_{{{{{{{{\rm{e}}}}}}}}}\left(\nabla \overrightarrow{{{{{{{{{\rm{u}}}}}}}}}_{{{{{{{{\rm{e}}}}}}}}}}+{\left(\nabla \overrightarrow{{{{{{{{{\rm{u}}}}}}}}}_{{{{{{{{\rm{e}}}}}}}}}}\right)}^{{{{{{{{\rm{T}}}}}}}}}\right)$$

where $\overrightarrow{{{{{{{\rm{u}}}}}}}_{{{{{{\rm{e}}}}}}}},\,{\mu }_{{{{{{\rm{e}}}}}}}$ and *ρ*_*e*_ are the velocity, viscosity, and the density of the electrolyte.

The velocity field of the gaseous mixture inside the GDE (CL+GDL) is approximated by Darcy’s law for porous media

6 $$\overrightarrow{{{{{{{{{\rm{u}}}}}}}}}_{{{{{{{{\rm{g}}}}}}}}}}=-\frac{{\kappa }_{{{{{{{{\rm{m}}}}}}}}}^{{{{{{{{\rm{eff}}}}}}}}}}{{\mu }_{{{{{{{{\rm{g}}}}}}}}}}\nabla {{{{{{{{\rm{p}}}}}}}}}_{{{{{{{{\rm{G}}}}}}}}}$$

where $\overrightarrow{{{{{{{\rm{u}}}}}}}_{{{{{{\rm{g}}}}}}}},{\mu }_{{{{{{\rm{g}}}}}}},{{{{{{\rm{p}}}}}}}_{{{{{{\rm{G}}}}}}}$ are the velocity, viscosity, and the pressure of the gaseous mixture. ${\kappa }_{{{{{{\rm{m}}}}}}}^{{{{{{{{\rm{eff}}}}}}}}}$ is the effective permeability of the medium m (m = CL or GDL), the details of which are given in Supplementary Methods 2 of the SI. For the cases with anisotropic CLs the permeability becomes a tensor. For the case with real CLs, properties have been obtained with pore-level simulations^21^.

The Reynolds number of the gas entering the gas flow channel is below 100 for all computed flow rates, hence a laminar compressible flow regime is assumed, resulting in ref. ^18^,

7 $$\nabla \cdot \left({\rho }_{{{{{{{{\rm{g}}}}}}}}}\overrightarrow{{{{{{{{{\rm{u}}}}}}}}}_{{{{{{{{\rm{g}}}}}}}}}}\right)=0$$

8 $${\rho }_{{{{{{{{\rm{g}}}}}}}}}\left(\overrightarrow{{{{{{{{{\rm{u}}}}}}}}}_{{{{{{{{\rm{g}}}}}}}}}}\cdot \nabla \overrightarrow{{{{{{{{{\rm{u}}}}}}}}}_{{{{{{{{\rm{g}}}}}}}}}}\right)=\nabla {{{{{{{{\rm{p}}}}}}}}}_{{{{{{{{\rm{G}}}}}}}}}+{\mu }_{{{{{{{{\rm{g}}}}}}}}}\left(\nabla \overrightarrow{{{{{{{{{\rm{u}}}}}}}}}_{{{{{{{{\rm{g}}}}}}}}}}+{\left(\nabla \overrightarrow{{{{{{{{{\rm{u}}}}}}}}}_{{{{{{{{\rm{g}}}}}}}}}}\right)}^{{{{{{{{\rm{T}}}}}}}}}\right)-\frac{2}{3}{\mu }_{{{{{{{{\rm{g}}}}}}}}}\left(\nabla \cdot \overrightarrow{{{{{{{{{\rm{u}}}}}}}}}_{{{{{{{{\rm{g}}}}}}}}}}\right)I$$

where $\overrightarrow{{u}_{g}},{\mu }_{g}$, and *ρ*_g_ are the velocity, viscosity, and the density of the gaseous mixture

##### Charge conservation equations

Charge conservation and Ohm’s law govern the electronic potential (Φ_s_) and the current density ($\overrightarrow{{{{{{{\rm{i}}}}}}}_{{{{{{{{\rm{s}}}}}}}}}}$), as

9 $$\nabla \cdot \overrightarrow{{{{{{{{{\rm{i}}}}}}}}}_{{{{{{{{\rm{s}}}}}}}}}}=-\nabla \cdot \overrightarrow{{{{{{{{{\rm{i}}}}}}}}}_{{{{{{{{\rm{l}}}}}}}}}}=-{{{{{{{{\rm{a}}}}}}}}}_{{{{{{{{\rm{v}}}}}}}}}\mathop{\sum}\limits_{{{{{{{{\rm{k}}}}}}}}}{{{{{{{{\rm{i}}}}}}}}}_{{{{{{{{\rm{k}}}}}}}}}$$

10 $$\overrightarrow{{{{{{{{{\rm{i}}}}}}}}}_{{{{{{{{\rm{s}}}}}}}}}}=-{\sigma }_{{{{{{{{\rm{s}}}}}}}},{{{{{{{\rm{m}}}}}}}}}^{{{{{{{{\rm{eff}}}}}}}}}\nabla {\Phi }_{{{{{{{{\rm{s}}}}}}}}}$$

where, a_v_ is the active specific surface area, i_k_ is the local partial current density for reaction ${{{{{\rm{k}}}}}},{\sigma }_{{{{{{{{\rm{s}}}}}}}},{{{{{{{\rm{m}}}}}}}}}^{{{{{{{{\rm{eff}}}}}}}}}$ is the effective electrical conductivity of the solid material in the medium m, corrected for the porosity and tortuosity (*τ*_m_) of the medium using the Bruggeman correlation,

11 $${\sigma }_{{{{{{{{\rm{s}}}}}}}},{{{{{{{\rm{m}}}}}}}}}^{{{{{{{{\rm{eff}}}}}}}}}={\sigma }_{{{{{{{{\rm{m}}}}}}}}}\frac{{\varepsilon }_{{{{{{{{\rm{m}}}}}}}}}}{{\tau }_{{{{{{{{\rm{m}}}}}}}}}}$$

For the cases with anisotropic CLs the effective conductivities become a tensor. For the case with real CLs, properties have been obtained with pore-level simulations^21^. Finally, charge conservation in the electrolyte phase is imposed through the electroneutrality condition,

12 $$\sum {{{{{{{{\rm{z}}}}}}}}}_{{{{{{{{\rm{i}}}}}}}}}{{{{{{{{\rm{c}}}}}}}}}_{{{{{{{{\rm{i}}}}}}}}}=0$$

##### Species conservation equations

Transport of each species (either in the gaseous phase or in the aqueous phase) can be written as follows,

13 $$\nabla \cdot \overrightarrow{{{{{{{{{\rm{n}}}}}}}}}_{{{{{{{{\rm{i}}}}}}}}}}={{{{{{{{\rm{R}}}}}}}}}_{{{{{{{{\rm{CT}}}}}}}},{{{{{{{\rm{i}}}}}}}}}+{{{{{{{{\rm{R}}}}}}}}}_{{{{{{{{\rm{B}}}}}}}},{{{{{{{\rm{i}}}}}}}}}+{{{{{{{{\rm{R}}}}}}}}}_{{{{{{{{\rm{PT}}}}}}}},{{{{{{{\rm{i}}}}}}}}}$$

where $\overrightarrow{{{{{{{\rm{n}}}}}}}_{{{{{{\rm{i}}}}}}}}$ is the species molar flux, R_CT,i_, R_B,i_, and R_PT,i_ are the volumetric source terms from charge-transfer reactions, homogeneous bulk reactions and phase-transfer reactions, respectively. Transport of gas-phase species (CO_2_, H_2_, and CO) takes place in the CL (only for I.W case), GDL and the gas-flow channel. The mass flux of j^th^ gaseous species ($\overrightarrow{{{{{{{\rm{n}}}}}}}_{{{{{{\rm{j}}}}}}}}$) consists of a diffusive term and a convective term,

14 $$\overrightarrow{{{{{{{{{\rm{n}}}}}}}}}_{{{{{{{{\rm{j}}}}}}}}}}=-\overrightarrow{{{{{{{{{\rm{j}}}}}}}}}_{{{{{{{{\rm{j}}}}}}}}}}+{\rho }_{{{{{{{{\rm{j}}}}}}}}}\overrightarrow{{{{{{{{{\rm{u}}}}}}}}}_{{{{{{{{\rm{g}}}}}}}}}}$$

The diffusive flux (j_j_) is calculated using a mixture averaged diffusion model^30^, the details of which are mentioned in Supplementary Methods 3 of the SI.

The molar flux of the i^th^ electrolytic species (n_i_) (i = ${{{{{{{{\rm{CO}}}}}}}}}_{2({{{{{{{\rm{aq}}}}}}}})},{{{{{{{{\rm{HCO}}}}}}}}}_{{3}^{-}},{{{{{{{{\rm{CO}}}}}}}}}_{3}^{2-},{{{{{{{{\rm{OH}}}}}}}}}^{-},{{{{{{{{\rm{K}}}}}}}}}^{+},{{{{{{{{\rm{H}}}}}}}}}^{+},{{{{{{{\rm{CO}}}}}}}},{{{{{{{{\rm{H}}}}}}}}}_{2}$) in the electrolyte flow channel or the CL is given by Nernst-Plank equation as follows,

15 $$\overrightarrow{{{{{{{{{\rm{n}}}}}}}}}_{{{{{{{{\rm{i}}}}}}}}}}=-{{{{{{{{\rm{D}}}}}}}}}_{{{{{{{{\rm{i}}}}}}}}}\nabla {{{{{{{{\rm{c}}}}}}}}}_{{{{{{{{\rm{i}}}}}}}}}+{{{{{{{{\rm{z}}}}}}}}}_{{{{{{{{\rm{i}}}}}}}}}{{{{{{{\rm{F}}}}}}}}\frac{{{{{{{{{\rm{D}}}}}}}}}_{{{{{{{{\rm{i}}}}}}}}}}{{{{{{{{\rm{RT}}}}}}}}}{{{{{{{{\rm{c}}}}}}}}}_{{{{{{{{\rm{i}}}}}}}}}\nabla {\Phi }_{{{{{{{{\rm{l}}}}}}}}}+{{{{{{{{\rm{c}}}}}}}}}_{{{{{{{{\rm{i}}}}}}}}}\overrightarrow{{{{{{{{{\rm{u}}}}}}}}}_{{{{{{{{\rm{e}}}}}}}}}}$$

For the transport of aqueous species in the porous CL, the diffusivities are corrected using the Bruggeman relationship and the convective term is not considered. For the cases with anisotropic CLs the effective diffusivities become a tensor. For the case with real CLs, properties have been obtained with pore-level simulations^21^.

#### Reactions

##### Electrochemical reactions

The two dominant electrochemical reactions occurring on the surface of the silver nano-particle are the carbon monoxide evolution reaction (COER) and the hydrogen evolution reaction (HER) given by the following equations

16 $${{{{{{{{\rm{CO}}}}}}}}}_{2({{{{{{{\rm{aq}}}}}}}}.)}+{{{{{{{{\rm{H}}}}}}}}}_{2}{{{{{{{\rm{O}}}}}}}}+2{{{{{{{{\rm{e}}}}}}}}}^{-}\longrightarrow {{{{{{{\rm{CO}}}}}}}}+2{{{{{{{{\rm{OH}}}}}}}}}^{-}$$

17 $$2{{{{{{{{\rm{H}}}}}}}}}_{2}{{{{{{{\rm{O}}}}}}}}+2{{{{{{{{\rm{e}}}}}}}}}^{-}\longrightarrow {{{{{{{{\rm{H}}}}}}}}}_{2}+2{{{{{{{{\rm{OH}}}}}}}}}^{-}$$

The COER and HER contribute to the source term for aqueous phase species (H_2_, CO, CO_2_(aq), OH^−^) in the CL through the Faraday’s law,

18 $${{{{{{{{\rm{R}}}}}}}}}_{{{{{{{{\rm{CT}}}}}}}},{{{{{{{\rm{i}}}}}}}}}=-\mathop{\sum}\limits_{{{{{{{{\rm{k}}}}}}}}}\frac{{{{{{{{{\rm{s}}}}}}}}}_{{{{{{{{\rm{i}}}}}}}},{{{{{{{\rm{k}}}}}}}}}{{{{{{{{\rm{a}}}}}}}}}_{{{{{{{{\rm{v}}}}}}}}}{{{{{{{{\rm{i}}}}}}}}}_{{{{{{{{\rm{k}}}}}}}}}}{{{{{{{{{\rm{n}}}}}}}}}_{{{{{{{{\rm{k}}}}}}}}}{{{{{{{\rm{F}}}}}}}}}$$

where F is Faraday’s constant, n_k_ is the number of electrons transferred in the reaction k, s_i,k_ is the stoichiometric coefficient (negative for reactants and positive for products) for species i in reaction k and i_k_ is the partial current density of reaction k. a_v_ is the active specific surface area, defined as the total surface area of the catalyst nano-particles covered by the electrolyte to the total volume of the catalyst layer. It is given by the expression^15^,

19 $${{{{{{{{\rm{a}}}}}}}}}_{{{{{{{{\rm{v}}}}}}}}}={{{{{{{{\rm{a}}}}}}}}}_{{{{{{{{\rm{v}}}}}}}}}^{{{{{{{{\rm{o}}}}}}}}}{{{{{{{\rm{S}}}}}}}}$$

where S is the saturation parameter defined as the total electrolyte volume in the CL to the total pore volume of the CL and ${a}_{{{{{{{{\rm{v}}}}}}}}}^{o}$ is the specific surface area defined as the total surface area of the catalyst nano-particles to the total volume of the catalyst layer and is given by the expression,

20 $${{{{{{{{\rm{a}}}}}}}}}_{{{{{{{{\rm{v}}}}}}}}}^{{{{{{{{\rm{o}}}}}}}}}=\frac{3\left(1-{\varepsilon }_{{{{{{{{\rm{CL}}}}}}}}}^{{{{{{{{\rm{o}}}}}}}}}\right)}{{{{{{{{{\rm{r}}}}}}}}}_{{{{{{{{\rm{np}}}}}}}}}}$$

where r_np_ is the radius of the catalyst nano-particle and ${\varepsilon }_{{{{{{{{\rm{CL}}}}}}}}}^{{{{{{{{\rm{o}}}}}}}}}$ is the intrinsic porosity of the CL. For the case with real CLs, morphological properties have been obtained with pore-level simulations^21^.

The partial current density of COER is modeled using concentration-dependent Tafel equation (Eq. (21)), while the partial current density of HER is modeled using concentration-independent Tafel equation (Eq. (22)), respectively. In the scenario that we have considered (pH = 8.55 of the bulk electrolyte), the CL environment is highly alkaline for all the operating conditions considered. In such alkaline conditions, the only major source for hydrogen evolution is considered to be water reduction.

21 $${{{{{{{{\rm{i}}}}}}}}}_{{{{{{{{\rm{CO}}}}}}}}}=-{{{{{{{{\rm{i}}}}}}}}}_{{{{{{{{\rm{o}}}}}}}},{{{{{{{\rm{COER}}}}}}}}}\left(\frac{{{{{{{{{\rm{c}}}}}}}}}_{{{{{{{{{\rm{CO}}}}}}}}}_{2}({{{{{{{\rm{aq}}}}}}}}.)}}{{{{{{{{{\rm{c}}}}}}}}}_{{{{{{{{{\rm{CO}}}}}}}}}_{2}({{{{{{{\rm{aq}}}}}}}}.)}^{{{{{{{{\rm{ref}}}}}}}}}}\right)\exp \left(-\frac{{\alpha }_{{{{{{{{\rm{c}}}}}}}},{{{{{{{\rm{COER}}}}}}}}}{{{{{{{\rm{F}}}}}}}}}{{{{{{{{\rm{RT}}}}}}}}}{\eta }_{{{{{{{{\rm{s}}}}}}}},{{{{{{{\rm{COER}}}}}}}}}\right)$$

22 $${{{{{{{{\rm{i}}}}}}}}}_{{{{{{{{{\rm{H}}}}}}}}}_{2}}=-{{{{{{{{\rm{i}}}}}}}}}_{{{{{{{{\rm{o}}}}}}}},{{{{{{{\rm{HER}}}}}}}}}\exp \left(-\frac{{\alpha }_{{{{{{{{\rm{c}}}}}}}},{{{{{{{\rm{HER}}}}}}}}}{{{{{{{\rm{F}}}}}}}}}{{{{{{{{\rm{RT}}}}}}}}}{\eta }_{{{{{{{{\rm{s}}}}}}}},{{{{{{{\rm{HER}}}}}}}}}\right)$$

Here, i_o,k_ and α_c,k_ are the exchange current density and charge-transfer coefficient for reaction k, respectively. For convenience, the reference concentration of CO_2_ is arbitrarily taken to be 1M^15^. The surface overpotential for reaction k (*η*_s,k_) measured on the standard hydrogen electrode (SHE) scale is given by

23 $${\eta }_{{{{{{{{\rm{s}}}}}}}},{{{{{{{\rm{k}}}}}}}}}=\left({\Phi }_{{{{{{{{\rm{s}}}}}}}}}-{\Phi }_{1}\right)-\left({{{{{{{{\rm{U}}}}}}}}}_{{{{{{{{\rm{k}}}}}}}}}^{{{{{{{{\rm{o}}}}}}}}}-\frac{2.303{{{{{{{\rm{RT}}}}}}}}}{{{{{{{{\rm{F}}}}}}}}}{{{{{{{\rm{pH}}}}}}}}\right)$$

where Φ_s_ is the electrode potential measured against a SHE reference. ${{{{{{\rm{U}}}}}}}_{{{{{{\rm{k}}}}}}}^{{{{{{{{\rm{o}}}}}}}}}$ is the equilibrium potential of reaction k measured against a RHE reference, and the $-\frac{2.303{{{{{\rm{RT}}}}}}}{{{{{{\rm{F}}}}}}}{{{{{{{\rm{pH}}}}}}}}$ term is invoked to shift the equilibrium potentials from RHE to the SHE scale via the Nernst equation using local pH values^15^.

##### Homogeneous reactions

The homogeneous reactions occurring in the electrolyte are the acid/base carbonate and water-dissociation reactions, given by:

24 $${{{{{{{{\rm{CO}}}}}}}}}_{2({{{{{{{\rm{aq}}}}}}}}.)}+{{{{{{{{\rm{H}}}}}}}}}_{2}{{{{{{{\rm{O}}}}}}}}\leftrightarrow {{{{{{{{\rm{H}}}}}}}}}^{+}+{{{{{{{{\rm{HCO}}}}}}}}}_{3}^{-}$$

25 $${{{{{{{{\rm{HCO}}}}}}}}}_{3}^{-}\leftrightarrow {{{{{{{{\rm{H}}}}}}}}}^{+}+{{{{{{{{\rm{CO}}}}}}}}}_{3}^{2-}$$

26 $${{{{{{{{\rm{CO}}}}}}}}}_{2({{{{{{{\rm{aq}}}}}}}}.)}+{{{{{{{{\rm{OH}}}}}}}}}^{-}\leftrightarrow {{{{{{{{\rm{HCO}}}}}}}}}_{3}^{-}$$

27 $${{{{{{{{\rm{HCO}}}}}}}}}_{3}^{-}+{{{{{{{{\rm{OH}}}}}}}}}^{-}\leftrightarrow {{{{{{{{\rm{H}}}}}}}}}_{2}{{{{{{{\rm{O}}}}}}}}+{{{{{{{{\rm{CO}}}}}}}}}_{3}^{2-}$$

28 $${{{{{{{{\rm{H}}}}}}}}}_{2}{{{{{{{\rm{O}}}}}}}}\leftrightarrow {{{{{{{{\rm{H}}}}}}}}}^{+}+{{{{{{{{\rm{OH}}}}}}}}}^{-}$$

These reactions give rise to source terms for all aqueous phase species (except K^+^) through the following kinetic expression,

29 $${{{{{{{{\rm{R}}}}}}}}}_{{{{{{{{\rm{B}}}}}}}},{{{{{{{\rm{i}}}}}}}}}={\epsilon }_{{{{{{{{\rm{CL}}}}}}}}}^{{{{{{{{\rm{o}}}}}}}}}{{{{{{{\rm{S}}}}}}}}\mathop{\sum}\limits_{{{{{{{{\rm{n}}}}}}}}}{{{{{{{{\rm{s}}}}}}}}}_{{{{{{{{\rm{i}}}}}}}},{{{{{{{\rm{n}}}}}}}}}\left({{{{{{{{\rm{k}}}}}}}}}_{{{{{{{{\rm{n}}}}}}}}}\mathop{\prod}\limits_{{{{{{{{{\rm{s}}}}}}}}}_{{{{{{{{\rm{i}}}}}}}},{{{{{{{\rm{n}}}}}}}}} < 0}{{{{{{{{\rm{a}}}}}}}}}_{{{{{{{{\rm{i}}}}}}}}}^{-{{{{{{{{\rm{s}}}}}}}}}_{{{{{{{{\rm{i}}}}}}}},{{{{{{{\rm{n}}}}}}}}}}-\frac{{{{{{{{{\rm{k}}}}}}}}}_{{{{{{{{\rm{n}}}}}}}}}}{{{{{{{{{\rm{K}}}}}}}}}_{{{{{{{{\rm{n}}}}}}}}}}\mathop{\prod}\limits_{{{{{{{{{\rm{s}}}}}}}}}_{{{{{{{{\rm{i}}}}}}}},{{{{{{{\rm{n}}}}}}}}} > 0}{{{{{{{{\rm{a}}}}}}}}}_{{{{{{{{\rm{i}}}}}}}}}^{{{{{{{{{\rm{s}}}}}}}}}_{{{{{{{{\rm{i}}}}}}}},{{{{{{{\rm{n}}}}}}}}}}\right)$$

where, k_n_ and K_n_ are the forward rate and equilibrium constants of reaction n, respectively. a_i_ is the activity of species i, which is assumed to be same as the species concentration.

#### Phase-transfer reactions

##### Ideally wetted CL case

CO_2_ phase-transfer reaction in the CL gives rise to source terms for both, gaseous and aqueous-phase CO_2_, through

30 $${{{{{{{{\rm{R}}}}}}}}}_{{{{{{{{\rm{PT}}}}}}}},{{{{{{{{\rm{CO}}}}}}}}}_{2}}={{{{{{{{\rm{a}}}}}}}}}_{{{{{{{{\rm{v}}}}}}}}}{{{{{{{{\rm{k}}}}}}}}}_{{{{{{{{\rm{GL}}}}}}}},{{{{{{{{\rm{CO}}}}}}}}}_{2}}\left({{{{{{{{\rm{H}}}}}}}}}_{{{{{{{{{\rm{CO}}}}}}}}}_{2}}{{{{{{{{\rm{p}}}}}}}}}_{{{{{{{{\rm{G}}}}}}}}}{{{{{{{{\rm{x}}}}}}}}}_{{{{{{{{{\rm{CO}}}}}}}}}_{2}}-{{{{{{{{\rm{c}}}}}}}}}_{{{{{{{{{\rm{CO}}}}}}}}}_{2}({{{{{{{\rm{aq}}}}}}}})}\right)$$

where ${{{{{{\rm{R}}}}}}}_{{{{{{{{\rm{PT}}}}}}}},{{{{{{{{\rm{CO}}}}}}}}}_{2}}$ is negative for gas-phase CO_2_ and positive for liquid-phase CO_2_. ${{{{{{\rm{k}}}}}}}_{{{{{{{{\rm{GL}}}}}}}},{{{{{{{{\rm{CO}}}}}}}}}_{2}}$ is the gas-to-liquid mass-transfer coefficient dependent on the thickness of the electrolyte film covering the catalyst nano-particle (*δ*_TF_) and the species diffusivity^15^,

31 $${{{{{{{{\rm{k}}}}}}}}}_{{{{{{{{\rm{GL}}}}}}}},{{{{{{{{\rm{CO}}}}}}}}}_{2}}=\frac{{{{{{{{{\rm{D}}}}}}}}}_{{{{{{{{{\rm{CO}}}}}}}}}_{2}({{{{{{{\rm{aq}}}}}}}})}}{{\delta }_{{{{{{{{\rm{TF}}}}}}}}}}$$

The Sechenov (salting out) effect is incorporated to determine Henry’s constant for CO_2_ dissolution in the electrolyte (${{{{{{\rm{H}}}}}}}_{{{{{{{{{\rm{CO}}}}}}}}}_{2}}$) through the following relation^31^,

32 $${{{{{{{{\rm{H}}}}}}}}}_{{{{{{{{\rm{g}}}}}}}}}={{{{{{{{\rm{H}}}}}}}}}_{0,{{{{{{{\rm{g}}}}}}}}}\mathop{\prod}\limits_{i}1{0}^{-({{{{{{{{\rm{h}}}}}}}}}_{{{{{{{{\rm{i}}}}}}}}}+{{{{{{{{\rm{h}}}}}}}}}_{{{{{{{{\rm{g}}}}}}}}}){{{{{{{{\rm{c}}}}}}}}}_{{{{{{{{\rm{i}}}}}}}}}}$$

where H_0,g_ is Henry’s constant of the g^th^ gas (where g = CO_2_, CO, H_2_) dissolved in pure water at ambient conditions and h_i_ are the ion-specific and gas-specific constants for the Schumpe form of the solubility correction^32^, which are provided in Supplementary Table 2. No parameters are available for CO, so the nominal CO Henry’s constant is not modified.

##### Fully flooded CL case

In case of fully flooded CL model, the phase transfer reactions occur at the boundary of CL and GDL (boundary 7 in Fig. 11) and are implemented through the following two conditions

33 $${{{{{{{{\rm{c}}}}}}}}}_{{{{{{{{\rm{i}}}}}}}}}={{{{{{{{\rm{H}}}}}}}}}_{{{{{{{{\rm{i}}}}}}}}}{{{{{{{{\rm{p}}}}}}}}}_{{{{{{{{\rm{G}}}}}}}}}{{{{{{{{\rm{x}}}}}}}}}_{{{{{{{{\rm{i}}}}}}}}}$$

34 $$\overrightarrow{{{{{{{{{\rm{n}}}}}}}}}_{{{{{{{{\rm{i}}}}}}}}}}=\overrightarrow{{{{{{{{{\rm{n}}}}}}}}}_{{{{{{{{\rm{j}}}}}}}}}}$$

The subscript i and j stands for aqueous phase and gaseous phase species, respectively. Equation (33) specifies the fully saturated condition of aqueous phase species i (i = CO_2_, H_2_, CO) at the CL-GDL interface. Equation (34) equates the fluxes in the two phases for the three phase changing species.

### Numerical details

The governing equations and the boundary conditions are solved iteratively at a steady state using MUMPS general solver in COMSOL Multiphysics 6.0 with a relative tolerance of 0.001. A user-defined mesh with mapped distribution was used and the number of elements in each domain was varied to ensure a mesh-independent solution. EC, GDL, and GC are meshed horizontally with 50 elements each. These elements grow exponentially and symmetrically within their respective domains with a growth ratio of 10. On the other hand, CL is meshed using 100 symmetrically and exponentially growing elements with a growth ratio of 20. In the vertical direction, the electrode height is meshed using 200 elements that grow exponentially and symmetrically, with a growth ratio of 40. To enhance the convergence of the model, all the parametric sweeps were performed utilizing the solution of the previous step as an initial condition for the next step. This allows the use of a single steady-state solver which simultaneously solves the multi-physical non-linear processes occurring inside the GDE and the flow channels. We used different physics interfaces in COMSOL Multiphysics to solve for various variables of interest. The concentration of different aqueous species and the electrolyte potential were solved using the ‘Tertiary current distribution’ physics interface. For the mole fractions of gaseous species in the GDL and GC, we employed the ‘transport of concentrated species’ interface. To determine the velocity and pressure profiles of the electrolyte and gas flow in their respective channels, we utilized the ‘laminar flow’ physics module. Finally, the gas flow through the GDL was solved using the ‘Darcy’s law’ physics interface.

### Supplementary information

Supplementary Material

## Data Availability

The datasets generated and/or analyzed during the current study are available from the corresponding author on reasonable request.

## Code availability

The computational model is available upon reasonable request from the authors.
