# Supplementary material for: Quantifying mass transport limitations in a microfluidic CO2 electrolyzer with a gas diffusion cathode
Source: Commun Chem. 2024 Mar 5;7:47. doi: 10.1038/s42004-024-01122-5 (PMC10914812; doi:10.1038/s42004-024-01122-5)
Supplement: Supplementary file 1 — Supplementary Material [file 42004_2024_1122_MOESM1_ESM.pdf]

Supplementary Information

Supplementary Methods 1: 1D modelling methodology

This section provides the additional information required to model the system in 1D.

Supplementary Note 1: Schematic

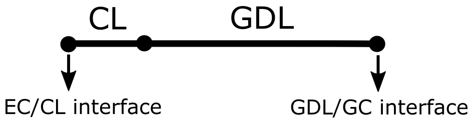

Supplementary Figure 1: Schematic representation of 1D GDE model.

Figure 1 displays a schematic of the 1D GDE model, showing two modeling domains, i.e., CL and GDL. Unlike the 2D GDE model, the flow channels for the electrolyte and the reactant CO_2 gas are excluded, and their effect is incorporated through the boundary conditions specified at the EC/CL and GDL/GC interface, respectively.

Supplementary Note 2: Boundary Conditions

The CO_2 mole fraction at the GDL/GC interface is specified as one and the mass-flux at the EC/CL interface is given by Eq. S1 for j^{th} electrolytic species.

$$\mathrm{n_j} = \rho_{\mathrm{liq}} \mathrm{k_{MT,j}} \left(\omega_{\mathrm{j}}^{\mathrm{b}} - \omega_{\mathrm{j}} \right) \tag{S1}$$

Here, k_{MT} is the mass-transfer coefficient calculated using Sherwood–Reynold–Schmidt correlation given by Eq. S2 [1]. ω_j^{b} and ω_j are the jth species mass-fractions in the bulk and at the EC/CL interface, respectively. This particular correlation was chosen to ensure our 1D model represents typical 1D models in the literature (specifically [1]). However, this correlation would need to be adapted if it were to be used for the analysis of an actual device.

$$\mathrm{k_{MT,j}} = 0.664 \left(\frac{\mathrm{D_j}}{\mathrm{H}} \right) \left(\frac{\rho_{\mathrm{liq}} \mathrm{u_{liq}} \mathrm{H}}{\mu_{\mathrm{liq}}} \right)^{1/2} \left(\frac{\mu_{\mathrm{liq}}}{\rho_{\mathrm{liq}} \mathrm{D_j}} \right)^{1/3} \tag{S2}$$

Here, u_{liq} is the velocity of the electrolyte at the inlet of the EC.

Supplementary Methods 2: Effective Permeability

κ_m^{eff} is the effective permeability of the medium m (m= CL or GDL) given by,

$$\kappa_{\text{m}}^{\text{eff}} = \kappa_{\text{sat,m}} \kappa_{\text{r,m}} \tag{S3}$$

where $\kappa_{\text{sat,m}}$ is the saturated permeability determined by the porosity (ε_{m}) and the fully saturated permeability of the medium ($\kappa_{\text{sat,m}}^{\text{o}}$) according to the following relationship [2]

$$\kappa_{\text{sat,m}} = \kappa_{\text{sat,m}}^{\text{o}} \frac{\varepsilon_{\text{m}}^3}{(1-\varepsilon_{\text{m}})^2} \tag{S4}$$

and $\kappa_{\text{r,m}}$ is the relative permeability which has a cubic dependence on the saturation (S) of the medium as follows [1, 3],

$$\kappa_{\text{r,m}} = (1 - \text{S})^3 \tag{S5}$$

The porosity of the medium m is given by,

$$\varepsilon_{\text{m}} = \varepsilon_{\text{m}}^{\text{o}}(1 - S) \tag{S6}$$

where $\varepsilon_{\text{m}}^{\text{o}}$ is the porosity of the medium m at $S = 0$.

Supplementary Methods 3: Mixture averaged diffusion model

The diffusive flux (j_j) given in Eq. 14 is calculated using a mixture averaged diffusion model [4]

$$\vec{\text{j}_{\text{j}}} = \rho_{\text{g}} \text{D}_{\text{j}}^{\text{eff}} \nabla \omega_{\text{j}} + \rho_{\text{g}} \text{D}_{\text{j}}^{\text{eff}} \omega_{\text{j}} \frac{\nabla \text{M}_{\text{n}}}{\text{M}_{\text{n}}} \tag{S7}$$

where ω_j is the species mass fraction, M_n is the average molar mass of the mixture ($M_n = \left(\sum_j \frac{\omega_j}{M_j}\right)^{-1}$), D_j^{eff} is the effective diffusion coefficient corrected using Bruggeman relationship,

$$\text{D}_{\text{j}}^{\text{eff}} = \text{D}_{\text{j}} \frac{\varepsilon_{\text{m}}}{\tau_{\text{m}}} \tag{S8}$$

The diffusion coefficient (D_j) is composed of a mass-averaged Stefan-Maxwell diffusivity (D_j^M) and Knudsen diffusivity (D_j^K), occurring in parallel as given by,

$$\frac{1}{\mathrm{D_j}} = \frac{1}{\mathrm{D_j^K}} + \frac{1}{\mathrm{D_j^M}} \tag{S9}$$

where,

$$\mathrm{D_j^K} = \frac{2\mathrm{r_{p,m}}}{3}\sqrt{\frac{8\mathrm{RT}}{\pi \mathrm{M_j}}} \tag{S10}$$

$$\mathrm{D_j^M} = \frac{1-\omega_\mathrm{j}}{\sum_{\mathrm{k}\neq\mathrm{j}} \frac{\mathrm{x_k}}{\mathrm{D_{jk}}}} \tag{S11}$$

Here, $r_{p,m}$ is the average pore radius of the porous medium m, x_j and M_j are the molar fraction and the molar weight of the jth species, respectively. Knudsen diffusivity is relevant primarily within the CL, but not in the GDL. This distinction arises because the pore radius within the GDL is significantly larger than the mean free path of the gas molecules, rendering Knudsen diffusion negligible. In contrast, within the CL, the two length scales (pore size and mean free path) are closer in magnitude, making it necessary to take Knudsen diffusivity into account when considering gas transport in the CL.

Finally, the mass-constraint condition is used to compute the mass-fraction of the Nth species as follows,

$$\sum_j \omega_j = 1 \tag{S12}$$

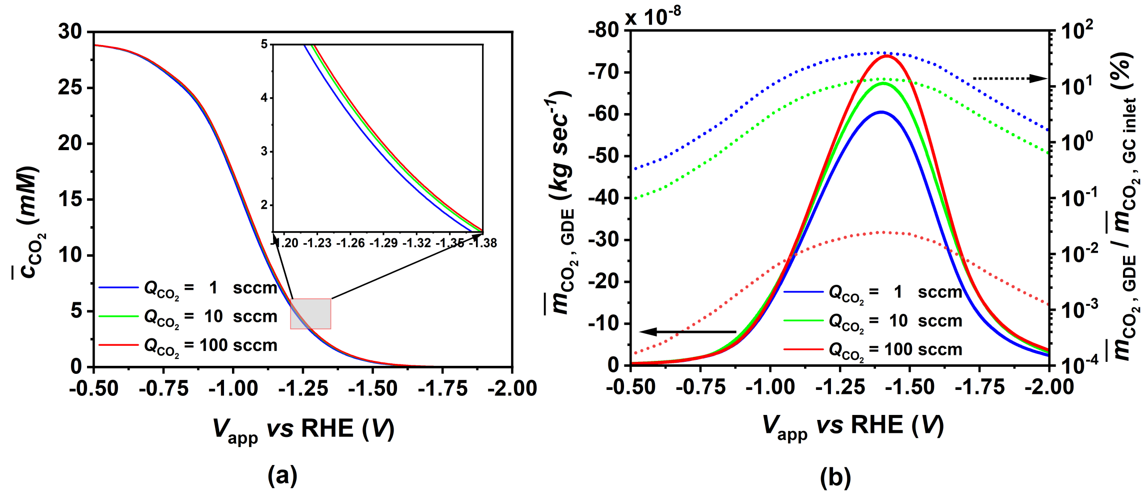

Supplementary Figure 2: (a) Averaged CO_2 concentration in the CL, (b) averaged gaseous CO_2 mass flow rate (x-component) at the GC/GDL interface in absolute terms (left y-axis) and as a percentage of averaged gaseous CO_2 mass flow rate at the GC inlet (right y-axis), as a function of applied cathode potential for different values of CO_2 gas flow rate at the GC inlet.

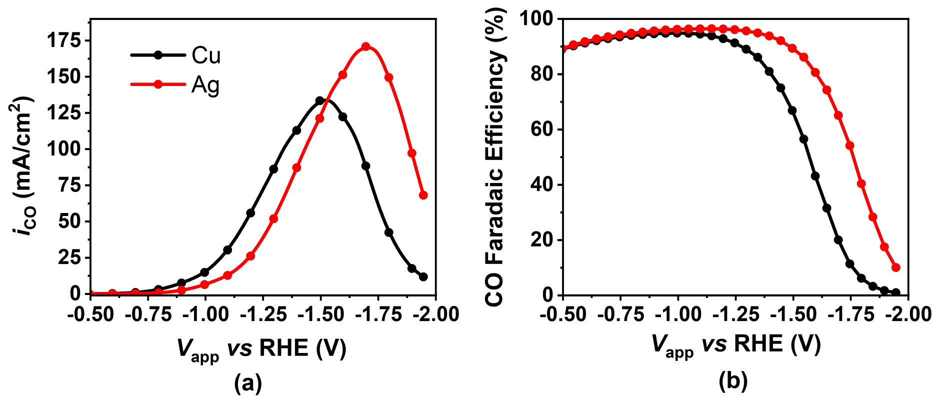

Supplementary Figure 3: (a) CO PCD and (b) CO Faradaic efficiency as a function of applied cathode potential for two different electrode materials (Cu and Ag).

Parameter	Description	Value	Unit	Ref.
L_{GC}	Thickness of the GC	5	mm	[5]
L_{EC}	Thickness of the EC	1.5	mm	[5]
L_{GDL}	Thickness of the GDL	325	μm	[6]
L_{CL}	Thickness of the CL	3.81	μm	[1]
$L_{\mathrm{GCext,lower}}$	Length of the extension at the lower end of GC	10	mm	
$L_{\mathrm{GCext,upper}}$	Length of the extension at the upper end of GC	2	mm	
L_{ECext}	Length of the extension at the two ends of EC	2	mm	
H	Height of the electrode	2	cm	[7]
W	Width of the electrode	5	cm	[7]
$A_{\mathrm{electrode}}$	Area of the electrode	10	cm^2	[7]
A_{GC}	Cross-sectional area of the GC	250	mm^2	
A_{EC}	Cross-sectional area of the EC	75	mm^2	

Supplementary Table 1: Model parameters corresponding to the geometry of the electrochemical half-cell.

Parameter	Description	Value	Units	Ref.
k_1	Forward rate constant (Eq. 24)	$3.71 \cdot 10^{-2}$	s^{-1}	[8]
k_2	Forward rate constant (Eq. 25)	59.44	s^{-1}	[8]
k_3	Forward rate constant (Eq. 26)	$2.23 \cdot 10^{3}$	$M^{-1}s^{-1}$	[8]
k_4	Forward rate constant (Eq. 27)	$1 \cdot 10^{8}$	$M^{-1}s^{-1}$	[9]
k_w	Forward rate constant (Eq. 28)	$1.4 \cdot 10^{-3}$	Ms^{-1}	[8]
K_1	Equilibrium constant (Eq. 24)	$10^{-6.35}$	M	[9]
K_2	Equilibrium constant (Eq. 25)	$10^{-10.33}$	M	[9]
K_w	Equilibrium constant (Eq. 28)	10^{-14}	M^2	[9]
d_l	Equivalent thickness of electrolyte thin film	10	nm	[1]
$H_{0,\mathrm{CO_2}}$	Henry's constant	34.2	mM atm^{-1}	[10]
$H_{0,\mathrm{CO}}$	Henry's constant	0.97	mM atm^{-1}	[10]
$H_{0,\mathrm{H_2}}$	Henry's constant	0.78	mM atm^{-1}	[10]
$h_{\mathrm{OH^-}}$	Salting out constant for OH^- ion	0.0839	M^{-1}	[10]
$h_{\mathrm{CO_3^{2-}}}$	Salting out constant for CO_3^{2-} ion	0.1423	M^{-1}	[10]
$h_{\mathrm{HCO_3^-}}$	Salting out constant for HCO_3^- ion	0.0967	M^{-1}	[10]
$h_{\mathrm{K^+}}$	Salting out constant for K^+ ion	0.0922	M^{-1}	[10]
$h_{\mathrm{CO_2}}$	Salting out constant for CO_2 gas	−0.172	M^{-1}	[10]
$h_{\mathrm{H_2}}$	Salting out constant for H_2 gas	−0.0218	M^{-1}	[10]
$i_{o,\mathrm{COER}}$	Exchange current density for COER	$4.71 \cdot 10^{-4}$	mA cm^{-2}	[1]
$i^B_{o,\mathrm{HER}}$	Exchange current density for HER in basic media	$1.16 \cdot 10^{-6}$	mA cm^{-2}	[1]
$i^A_{o,\mathrm{HER}}$	Exchange current density for HER in acidic media	$9.79 \cdot 10^{-4}$	mA cm^{-2}	[1]
α_{COER}	Charge transfer coefficient for COER	0.44		[1]
α^B_{HER}	Charge transfer coefficient for HER in basic media	0.36		[1]
α^A_{HER}	Charge transfer coefficient for HER in acidic media	0.27		[1]
U^o_{COER}	Standard reduction potential for COER vs RHE	-0.11	V	[11]
U^o_{HER}	Standard reduction potential for HER vs RHE	0	V	[11]

Supplementary Table 2: Model parameters for the different reactions.

Parameter	Description	Value	Units	Ref.
V_{app}	Applied potential at GDL/GC interface	-1 to -2.5	V(vs SHE)	
$Q_{\mathrm{gas,inlet}}$	Volumetric flow rate at the inlet of GC	17	sccm	[7]
$Q_{\mathrm{liq,inlet}}$	Volumetric flow rate at the inlet of EC	0.5	ml min^{-1}	[7]
$x_{\mathrm{CO_2}}$	CO_2 mole fraction at the inlet of the GC	1		[7]
$P_{\mathrm{gas,outlet}}$	Pressure at the outlet of GC	1	atm	
$P_{\mathrm{liq,outlet}}$	Pressure at the outlet of EC	1	atm	
$\mathrm{pH_b}$	pH of the bulk electrolyte (0.5 M $KHCO_3$)	8.55		[7]
$c_{b,\mathrm{HCO_3^-}}$	HCO_3^- concentration in the bulk electrolyte	0.483	M	[7]
$c_{b,\mathrm{CO_3^{2-}}}$	CO_3^{2-} concentration in the bulk electrolyte	8.03	mM	[7]
$c_{b,\mathrm{CO_2}}$	CO_2 concentration in the bulk electrolyte	3.05	mM	[7]
$c_{b,\mathrm{K^+}}$	K^+ concentration in the bulk electrolyte	0.5	M	[7]

Supplementary Table 3: Model parameters corresponding to cell operating conditions for the reference case.

Parameter	Description	Value	Unit	Ref
$D_{H_2,CO}$	Binary diffusion coefficient of H_2 in CO	0.743	$cm^2\ s^{-1}$	[12]
D_{H_2,CO_2}	Binary diffusion coefficient of H_2 in CO_2	0.646	$cm^2\ s^{-1}$	[12]
$D_{CO_2,CO}$	Binary diffusion coefficient of CO_2 in CO	0.152	$cm^2\ s^{-1}$	[12]
D_{K^+}	Diffusivity of K^+ ion in aqueous media	$1.957 * 10^{-5}$	$cm^2\ s^{-1}$	[12]
D_{H^+}	Diffusivity of H^+ ion in aqueous media	$9.311 * 10^{-5}$	$cm^2\ s^{-1}$	[12]
D_{OH^-}	Diffusivity of OH^- ion in aqueous media	$5.27 * 10^{-5}$	$cm^2\ s^{-1}$	[12]
$D_{HCO_3^-}$	Diffusivity of HCO_3^- ion in aqueous media	$1.18 * 10^{-5}$	$cm^2\ s^{-1}$	[12]
$D_{CO_3^{2-}}$	Diffusivity of CO_3^{2-} ion in aqueous media	$0.923 * 10^{-5}$	$cm^2\ s^{-1}$	[12]
D_{CO_2}	Diffusivity of CO_2 in aqueous media	$1.91 * 10^{-5}$	$cm^2\ s^{-1}$	[12]
D_{CO}	Diffusivity of CO in aqueous media	$2.03 * 10^{-5}$	$cm^2\ s^{-1}$	[12]
D_{H_2}	Diffusivity of H_2 in aqueous media	$4.875 * 10^{-5}$	$cm^2\ s^{-1}$	[12]
μ_{liq}	Viscosity of the electrolyte	$1.067 * 10^{-3}$	Pa.s	[12]
ρ_{liq}	Density of the electrolyte	1000	$kg\ m^{-3}$	[12]
μ_g	Viscosity of the gaseous mixture	$1.067 * 10^{-5}$	Pa.s	[12]

Supplementary Table 4: Model parameters corresponding to transport properties of chemical species.

Parameter	Description	Value	Units	Ref
$S_{\mathrm{I.W}}$	CL saturation for ideally wetted case	0.64		[1]
$S_{\mathrm{F.F}}$	CL saturation for fully flooded case	1.0		[1]
$\epsilon^{o}_{\mathrm{CL}}$	Porosity of the CL when S=0	0.5		[1]
ϵ_{CL}	Porosity of the CL	0.18		[1]
ϵ_{GDL}	Porosity of the GDL	0.8		[6]
σ_{CL}	Electrical conductivity of CL nanoparticles	100	S m^{-1}	[13]
σ_{GDL}	Electrical conductivity of GDL	220	S m^{-1}	[6]
r_{np}	Radius of CL nanoparticles	50	nm	[14]
$r_{\mathrm{p,CL}}$	Mean CL pore radius	25	nm	[1]
$r_{\mathrm{p,GDL}}$	Mean GDL pore radius	0.75	μm	[6]
m_{loading}	Catalyst loading	2	mg cm^{-2}	[7]
ρ_{Ag}	Density of the silver catalyst nanoparticle	10.49	g cm^{-3}	[1]
$a^{\mathrm{o}}_{\mathrm{v}}$	Specific surface area	$3 * 10^{7}$	m^{-1}	[1]
a_{v}	Active specific surface area	$1.9 * 10^{7}$	m^{-1}	[1]
κ_{CL}	Permeability of CL	$16 * 10^{-16}$	m^{2}	[15]
κ_{GDL}	Permeability of GDL	$1.34 * 10^{-12}$	m^{2}	[6]

Supplementary Table 5: Model parameters corresponding to material parameters of the GDE.

Sr No.	CL Property	Copper	Silver
1.	CL thickness (L_{CL})	1.5 μm	1.2 μm
2.	CL Porosity	0.9136	0.9815
3.	Radius of the np	42.24 nm	61.7 nm
4.	Roughness Factor (RF) (affects a_v=RF/L_{CL})	7.9	1
5.	CL diffusivity correction in x and y direction	0.698, 0.858	0.96,0.9785
6.	CL electronic conductivity correction in x and y direction	Bruggeman relationship	Bruggeman relationship

Supplementary Table 6: Material properties (transport and geometrical) of experimentally characterized Cu and Ag sample [16].

Supplementary References

[1] Weng, L.-C., Bell, A. T. & Weber, A. Z. Modeling gas-diffusion electrodes for co 2 reduction. *Physical Chemistry Chemical Physics* **20**, 16973–16984 (2018).

[2] Carman, P. C. Fluid flow through granular beds. *Chemical Engineering Research and Design* **75**, S32–S48 (1997).

[3] Moosavi, S. M., Niffeler, M., Gostick, J. & Haussener, S. Transport characteristics of saturated gas diffusion layers treated with hydrophobic coatings. *Chemical Engineering Science* **176**, 503–514 (2018).

[4] Taylor, R. & Krishna, R. *Multicomponent mass transfer*, vol. 2 (John Wiley & Sons, 1993).

[5] Jayashree, R. S., Mitchell, M., Natarajan, D., Markoski, L. J. & Kenis, P. J. Microfluidic hydrogen fuel cell with a liquid electrolyte. *Langmuir* **23**, 6871–6874 (2007).

[6] El-Kharouf, A., Mason, T. J., Brett, D. J. & Pollet, B. G. Ex-situ characterisation of gas diffusion layers for proton exchange membrane fuel cells. *Journal of Power sources* **218**, 393–404 (2012).

[7] Verma, S., Lu, X., Ma, S., Masel, R. I. & Kenis, P. J. The effect of electrolyte composition on the electroreduction of co 2 to co on ag based gas diffusion electrodes. *Physical Chemistry Chemical Physics* **18**, 7075–7084 (2016).

[8] Schulz, K. G., Riebesell, U., Rost, B., Thoms, S. & Zeebe, R. Determination of the rate constants for the carbon dioxide to bicarbonate inter-conversion in ph-buffered seawater systems. *Marine chemistry* **100**, 53–65 (2006).

[9] Gupta, N., Gattrell, M. & MacDougall, B. Calculation for the cathode surface concentrations in the electrochemical reduction of co2 in khco3 solutions. *Journal of applied electrochemistry* **36**, 161–172 (2006).

[10] Weisenberger, S. & Schumpe, d. A. Estimation of gas solubilities in salt solutions at temperatures from 273 k to 363 k. *AIChE Journal* **42**, 298–300 (1996).

[11] Blake, J., Padding, J. & Haverkort, J. Analytical modelling of co2 reduction in gas-diffusion electrode catalyst layers. *Electrochimica Acta* **393**, 138987 (2021).

[12] Newman, J. & Balsara, N. P. *Electrochemical systems* (John Wiley & Sons, 2021).

[13] Du, C., Shi, P., Cheng, X. & Yin, G. Effective protonic and electronic conductivity of the catalyst layers in proton exchange membrane fuel cells. *Electrochemistry communications* **6**, 435–440 (2004).

[14] Soboleva, T. *et al.* On the micro-, meso-, and macroporous structures of polymer electrolyte membrane fuel cell catalyst layers. *ACS applied materials & interfaces* **2**, 375–384 (2010).

[15] Zenyuk, I. V., Medici, E., Allen, J. & Weber, A. Z. Coupling continuum and pore-network models for polymer-electrolyte fuel cells. *International Journal of Hydrogen Energy* **40**, 16831–16845 (2015).

[16] Lorenzutti, F. & Haussener, S. Morphology and transport characterization of catalyst layers for co2 reduction. *Journal of The Electrochemical Society* (2023).
